# A seamlessly integrated device of micro-supercapacitor and wireless charging with ultrahigh energy density and capacitance

**DOI:** 10.1038/s41467-021-22912-8

**Published:** 2021-05-11

**Authors:** Chang Gao, Jiancheng Huang, Yukun Xiao, Guoqiang Zhang, Chunlong Dai, Zengling Li, Yang Zhao, Lan Jiang, Liangti Qu

**Affiliations:** 1 Beijing Key Laboratory of Photoelectronic/Electrophotonic Conversion Materials, School of Chemistry and Chemical Engineering, Beijing Institute of Technology, Beijing, PR China; 2 School of Microelectronics, Tianjin University, Tianjin, PR China; 3 Laser Micro-/Nano-Fabrication Laboratory, School of Mechanical Engineering, Beijing Institute of Technology, Beijing, PR China; 4 Key Laboratory of Organic Optoelectronics and Molecular Engineering of Ministry of Education, Department of Chemistry, Tsinghua University, Beijing, PR China

**Keywords:** Supercapacitors, Electrochemistry, Synthesis and processing

## Abstract

Microdevice integrating energy storage with wireless charging could create opportunities for electronics design, such as moveable charging. Herein, we report seamlessly integrated wireless charging micro-supercapacitors by taking advantage of a designed highly consistent material system that both wireless coils and electrodes are of the graphite paper. The transferring power efficiency of the wireless charging is 52.8%. Benefitting from unique circuit structure, the intact device displays low resistance and excellent voltage tolerability with a capacitance of 454.1 mF cm^−2^, superior to state-of-the-art conventional planar micro-supercapacitors. Besides, a record high energy density of 463.1 μWh cm^−2^ exceeds the existing metal ion hybrid micro-supercapacitors and even commercial thin film battery (350 μWh cm^−2^). After charging for 6 min, the integrated device reaches up to a power output of 45.9 mW, which can drive an electrical toy car immediately. This work brings an insight for contactless micro-electronics and flexible micro-robotics.

## Abstract (web summary)

Miniaturized energy storage devices integrated with wireless charging bring opportunities for next generation electronics. Here, authors report seamlessly integrated wireless charging micro-supercapacitors with high energy density capable of driving a model electrical car.

## Introduction

Miniaturized energy storage devices with flexibility and portability have become increasingly important in the development of next-generation electronics^1–5^. Generally, it still needs to find efficient connection ways for the energy storage microdevices and routine power supply equipment to complete the charging process, which runs counter to the original intention and future development of microdevices. To this end, replacing traditional electric supply mode with contactless charging can enhance the practicality of the energy storage microdevices in micro-drones, micro-electric vehicles, and micro-detective systems by eliminating the cumbersome circuit external connecting procedure.

Microdevices that combine energy storage and wireless charging functions can be defined as integrated wireless charging energy storage microdevices. In the current research stage, these wireless charging microdevices have been exploring as means to get rid of the unnecessary external connections and foreseeable physical electrode contact damage of microelectronic equipment, and to achieve high integration as well as high performance, realizing the multiple possibilities in advanced applications^6–9^. For example, in the early days, Gao^10^ adopted the ink-jet printing and magnetron sputtering method to obtain wireless charging coil (WCC) and micro-electrodes and assembled them into a flexible integrated wireless charging system to drive the photoconductive detector. Similarly, Akira^11^ utilized a laser-writing technology and electroless metal plating method to fabricate Ni-based WCCs and microdevices, in which the Ni-based coil and microdevices were directly connected by using copper tape. This integrated system exhibited a potential application in near-field communication. Later, Park^12^ further improved the integration of wireless charging microdevices by skillfully combining the wireless charging antenna and microdevices within the two-tier design, making the small-scale wirelessly rechargeable contact lenses possible.

It is worth noting that the wireless charging capability of the system is one of the critical factors that affect the overall energy of the microdevices, which strongly depends on the structure and electrical conductivity of coils inducing electromagnetic energy. Therefore, many natural metal and metal-based composites with highly received current have been widely applied in the current wireless charging microdevices in order to achieve high performance on the basis of integration. However, these metal-based coils are easy to oxidize and corrode, resulting in shortened lifetime^13–15^. Furthermore, their high heat loss will consume a large amount of electric energy, reducing practical utilization. Early studies on the wireless charging microdevices have also shown high energy consumption of joints and the vulnerable structural of the integrated microdevices due to the crude assembly of metal coils and electrodes. In theory, carbon materials with low cost, good stability, corrosion-resistant, and high conductivity may achieve similar performance to metal coils while overcoming their shortcomings^16^. However, the engineering realization of a high-performance metal-free coil in wireless charging micro-devices is hindered by the existing material systems due to the dearth of effective structural design and assembly protocols.

Micro-supercapacitors (MSCs) are particularly attractive in wireless charging storage microdevices because of their fast charging and discharging rate (adapting to changeable voltage), high power density (large driving force), and splendid cycling stability^17–21^. However, the state-of-art versions of wireless charging MSCs are difficult to be employed as wholeness devices in further practical applications because of the lack of suitable material engineering compatibility under the existing WCC mode^10,11^. Except for the reduction of integrity and flexibility, the energy output is also prone to be affected, not to mention acquiring possible breakthroughs in the high-capacity of wireless charging microdevices^22,23^.

In this work, we propose a kind of seamlessly integrated wireless charging MSCs (IWC-MSCs) by taking advantage of a designed high-consistent material system that wireless coils and electrodes are of the same material origin. Combined with the integrated circuit design, the IWC-MSCs could reach an intact structure, low current drain, and high voltage tolerability. In addition, the resulting IWC-MSCs with a remarkably high areal capacitance of 454.1 mF cm^−2^ are superior to the conventional planar MSCs reported previously. Apart from the wide potential range of 3 V, the IWC-MSCs device displays a record-high energy density of 463.1 μWh cm^−2^, even larger than the commercial thin-film batteries (350 μWh cm^−2^)^24^. Moreover, the IWC-MSCs could drive an electrical toy car with wireless charging of only 6 min, indicating the great potential in practical applications.

## Results

### Design and fabrication of IWC-MSCs

**Fig. 1:**
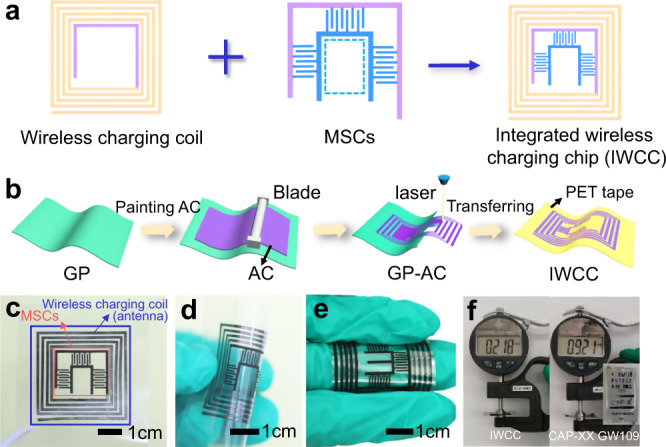
The design, fabrication, and electronic photos of IWC-MSCs. **a** Combination of WCC and MSCs in the integrated device, IWC-MSCs. **b** The schematic illustration of fabricating the integrated device. **c** The optical image of the integrated device IWC-MSCs. **d** Flexibility demonstration of the integrated device IWC-MSCs at a highly folded state. **e** The optical image of the integrated IWC-MSCs wrapping around one finger. **f** Thickness comparison of the IWC-MSCs with the thin-type commercial capacitor (CAP-XX GW109). The inset is the top view of the CAP-XX GW109. IWCC means the integrated wireless charging chip.

The IWC-MSCs device consists of two modules, the MSCs and the WCC acting as antennas (Fig. 1a). To integrate the two parts, we propose a planar coaxial structure, in which the MSCs are in the center surrounded by a coil. A shared electrode (purple line in Fig. 1a) is designed to connect the MSCs and WCC. Interestingly, we find that the shared electrode can not only act as a conductive line of the coil to harvest energy from the magnetic field but also use as an electrode of three interdigital parallel MSCs (blue lines in Fig. 1a).

Here, the number of MSCs is three instead of filling the center of IWC-MSCs. This is because that the MSCs in IWC-MSCs are inevitably affected by the magnetic field in the current system. According to Faraday’s law of *Electromagnetic induction*, if all the four sides of a square (blue dotted line in Fig. 1a) are used as electrodes of MSC, a new closed coil will be generated, which causes a similar induced magnetic field as the peripheral WCCs. It will affect the potential difference of the electrodes and thus makes an impact on the charge storage of the MSCs. As a result, the designed pattern of IWC-MSCs not only enables MSCs with the ability of wireless charging but also efficiently integrates WCC and MSCs in one device with a robust and intact structure.

In conventional WCC, the resistance of the coil is generally required to be lower than 1 Ω/m during transport of significant current in actual antenna^16^. However, most of the existing WCCs are limited to metal-based materials, which are easily corroded under harsh chemical conditions (e.g., acid environment, Supplementary Fig. 1a, b)^13–15^. To ensure the high integrity of IWC-MSCs, there is a great need to explore the materials that can be used for both electrodes and coils. Graphite paper (GP) has been widely used as circuit or electrode material^25^, due to its low sheet resistance (0.015 Ω/m), high conductivity, splendid corrosion resistance (Supplementary Fig. 1c, d), low heat generation (Supplementary Fig. 2), and high operating voltage (~3 V). Even in a sealed environment, the GP coil is more stable as one electrode in the IWC-MSCs while working in the electrolyte compared with the metal-based antenna (such as copper antenna, Supplementary Fig. 3). To this end, we propose that the GP is an ideal candidate for both WCC and electrode current collectors of MSCs.

Figure 1b demonstrates the fabricating process of the integrated device. A commercial GP (~50 μm) was used as the supporting and conductive substrate for MSC electrodes. The activated carbon (AC) mixed with poly(vinylidene fluoride) (PVDF), Ketjen black, and N-methyl pyrrolidone (NMP) that forming electrode slurry were then homogeneously painted on the GP with a blade to concisely control the thickness of the electrode. Here, PVDF, Ketjen black, and NMP were used as the binder, conductive particles, and solvent, respectively. After drying for 24 h in a vacuum at 60 °C, the GP coated with a layer of AC (GP–AC) was obtained. Thereafter, an integrated device with an organized graphic was ingeniously designed and manufactured by a simple one-step laser etching process on the GP–AC substrate. Finally, the patterned integrated device was transferred onto transparent PET tape to make sure the device is robust and steady. As shown in Fig. 1c, the integrated IWC-MSCs device consists of three interdigital MSCs connected in parallel (the center of the chip) and WCC (the outside of the chip) with a certain number of turns, in which the numbers of interdigital electrodes and coils are easily controlled by the laser etching machine in demands. Interestingly, the coil of the IWC-MSCs is constructed by extending one of the electrodes in MSCs to be as the antenna of wireless charging. This designed pattern subtly enables MSCs with the ability of wireless charging from the magnetic field, and efficiently shortens the connecting distance of different components, generating a low resistance of the whole device (Supplementary Fig. 4). Moreover, the integrated device with planarity feature presents good flexibility and maintains stable electrochemical properties under bending state at 90° (Fig. 1d, e, Supplementary Figs. 5 and 6, and Supplementary Movie 1). Notably, the integrated device exhibits a thickness of 0.218 mm (Fig. 1f), which is almost one-quarter of a thin-profile commercial CAP-XX GW109 capacitor (right, 0.921 mm), showing great potential in portable and ultrathin electronics. Besides, the size of the integrated device is also controllable which can be as small as ~1 cm^2^ footprint and even smaller under the high precision laser machine (Supplementary Fig. 7). Scanning electron microscope (SEM) image of interdigital electrodes of MSCs is shown in Supplementary Fig. 8, where a homogeneous AC layer attaches firmly on GP, and the electrodes have a clear and smooth edge, indicating a total-etch of laser writing. Both the gap and the width of the electrodes are 400 μm.

### Electrochemical characteristics of MSC

**Fig. 2:**
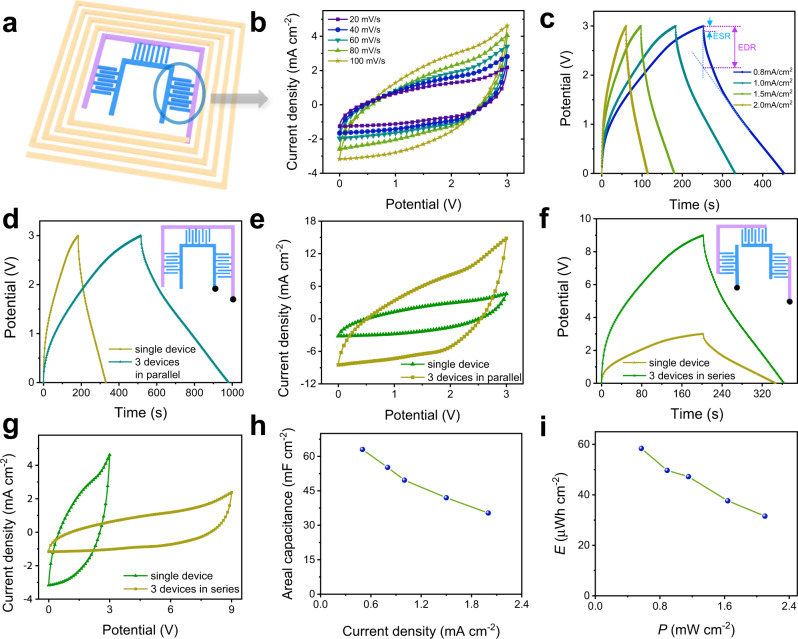
Electrochemical properties of a single MSC in the integrated device. **a** The scheme of one MSC in the integrated device. **b** CV curves measured at scan rates of 20–100 mV s^−1^. **c** The galvanostatic charging/discharging curves of a single MSC with different current densities. **d** Galvanostatic charging/discharging curves of a group of three MSCs connected in parallel. The inset is the schematic diagram of parallel connecting details, where black dots are testing points. **e** CV curves of MSCs connected in parallel. **f** Galvanostatic charging/discharging curves of a group of three MSCs connected in series. The inset is the schematic diagram of serial connecting details, and the black dots are testing points. **g** CV curves of MSCs connected in series. **h** The specific capacitance of a single MSC calculated from the discharging time under different currents. **i** Power and energy densities of a single MSC unit. *E* and *P* represent energy density and power density, respectively.

The electrochemical characterizations of a single MSC unit are investigated to evaluate its electrochemical performance (Fig. 2a). The ionic liquid of 1-ethyl-3-methylimidazolium bis (trifluoromethylsulfonimide) ([EMIM][TFSI]) with a high molar conductivity (2.3 cm^2^/Ωmol) and wide electrochemical window (~4.0 V) is selected as the electrolyte^26,27^. Besides, the [EMIM][TFSI] can also provide a high dielectric constant in double-layer capacitance of AC electrodes, contributing to store charges at the interface^28^. In order to avoid the impact of the air factor and leakage of liquid electrolyte, the MSC is sealed with PET tape. As shown in Fig. 2b, the cyclic voltammetry (CV) curves of the MSC present typical capacitive behaviors at different scanning rates of 20–100 mV/s^1,29^. Subtle redox peak may ascribe to a small part of the insertion of [EMIM]^+^ into graphite layers at ~2.2 V^30^. Since the [EMIM]^+^ intercalation in the graphite layers is unstable after charging and would decompose rapidly at an open circuit, both the CV and charging/discharging curves no longer remain ideal symmetry shape (Fig. 2b, c)^31,32^. Besides, the nonlinear of the discharge curves in Fig. 2c are attributed to the equivalent distributed resistance (EDR) at the beginning of discharging process, which consists of equivalent series resistance (ESR) and additional resistance caused by charge redistribution in the pores of electrode^33^. The ESR is mainly determined by the contact resistances, including contact between individual AC particles adhered by binders, the connection between activated carbon layer and GP, and the joints of copper foil and MSCs electrodes. Nevertheless, the charging/discharging curves show the near triangle shape, demonstrating the dominant electrical double layer capacitance behavior. The IR drop of the charging/discharging curves is 5.5% of the whole potential window at a low inert resistance of 94.1 Ω (Supplementary Fig. 9)^29,34^.

For further application of the MSC, we connect the MSCs in parallel and series. It can be observed that the charging and discharging time of the curves of the parallel device is three times of a single MSC (Fig. 2d), and the area of the enclosed curves of the parallel is almost three times of the single MSC (Fig. 2e). Besides, the MSCs connected in series exhibit a potential window three times higher than a single MSC (Fig. 2f, g), suggesting ideal tandem and parallel behavior of the fabricated MSCs. Figure 2h reveals the areal capacitance of a single MSC calculated according to the current density. At a current density of 0.5 mA cm^−2^, the areal capacitance has a high areal capacitance of 63 mF cm^−2^. Even if the current density increases to 2 mA cm^−2^, the areal capacitance reaches 35.3 mF cm^−2^, indicating good rate performance of the MSC. In accordance with the areal capacitance, the power density and energy density are obtained (Fig. 2i). It delivers energy density of 58.4 and 31.6 μWh cm^−2^ at the power density of 0.57 and 2.1 mW cm^−2^, respectively.

### Optimization and characterization of MSCs

**Fig. 3:**
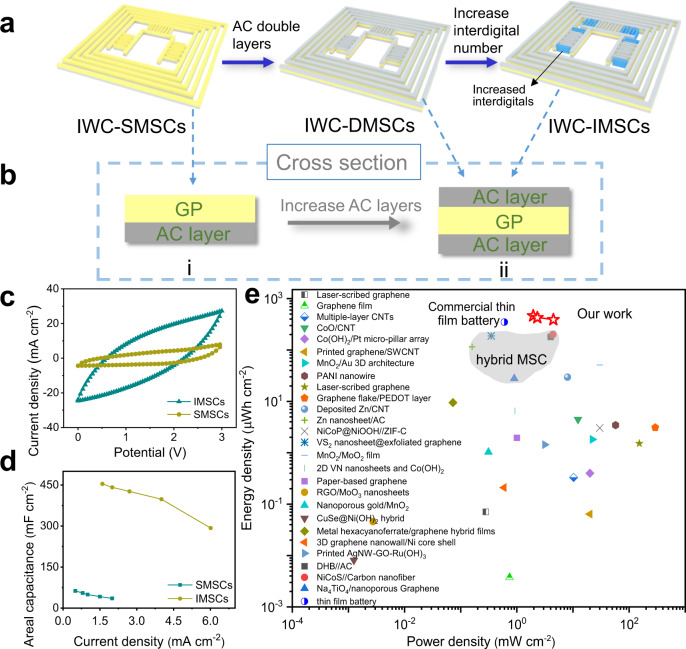
Process and measurements of improving the performance of MSCs in a device. **a** Schematic diagrams of the procedure of optimizing MSCs in IWC-MSCs. **b** Change of cross-section of SMSCs, DMSCs, and IMSCs. **c** Comparison of CV curves under the scanning rate of 100 mV/s and (**d**) rate capacity curves of MSCs of SMSCs and IMSCs, respectively. **e** Ragone plots of the IMSCs compared with other reported MSCs.

For practical use, there is a great need to increase the capacitance in a one-unit device to satisfy the demand for high-power electronics. An IWC-MSCs device consists of three parallel MSCs, which is viewed as an integral device for measurement. To this end, we find that the capacitance of the integrated MSC could be further improved by simply doubling the AC electrodes layer on both sides of the GP substrate without additional redundant devices (Fig. 3a, b). As shown in Fig. 3a, the structure of the integrated MSCs device changes from a single layer to double layers after introducing extra AC electrodes on the other side of GP. The corresponding devices are named single-layered MSCs (SMSCs) and double-layered MSCs (DMSCs), respectively. Figure 3b reveals the crossing section diagrams of IWC-SMSCs (i) and IWC-DMSCs (ii). The single layer of AC material on the GP substrate possesses a thickness of ~50 μm (Supplementary Fig. 10a). While the double layer has a similar thickness of ~50 μm on each side of the GP substrate (Supplementary Fig. 10b). The effect of thickness of the AC layer on performance is discussed in Supplementary Figs. 11 and 12. All the AC layers are closely in contact with the GP substrate. Moreover, the capacitance capability of the double-layered integrated MSCs can be further optimized by increasing the interdigital numbers (blue color in Fig. 3a) without changing the structure and shape of the device, and the improved MSCs are named as IMSCs. Besides, the effect of the gap between two adjacent interdigital electrodes is also discussed in Supplementary Fig. 13.

In order to ensure the stability in the air and isolate the interference of air and water on ionic liquid (Supplementary Figs. 14 and 15), the entire IMSCs device is encapsulated with PET tape before the test (the encapsulation details are shown in methods). All the electrochemical measurements of the IMSCs device are carried out in an air ambient environment. As shown in Supplementary Fig. 16a, the CV curve area of the IMSCs device increases with the scan rates from 20 to 200 mV/s, which is almost two times the enclosed area for the SMSCs (Fig. 3c), indicating the strong electrochemical storage ability. According to the galvanostatic charging/discharging curves under various current densities, a group of areal capacitances of IMSCs is calculated (Fig. 3d and Supplementary Fig. 16b). Clearly, the IMSCs exhibit a predominant larger areal capacitance advantage over the full range current densities than SMSCs (Fig. 3d). The areal capacitance of the IMSCs based on carbon material electrodes reaches an ultrahigh and maximum value of 430 mF cm^−2^ calculated by the integrated area of CV curves in Supplementary Fig. 16a and 454.1 mF cm^−2^ calculated based on the discharging curve at the current density of 1.6 mA cm^−2^ in Supplementary Fig. 16b (the areal capacitance is 171.7 mF cm^−2^ if the gap of electrodes is included), far superior to those MSCs reported previously (Supplementary Table 1). Calculation of electrodes area is demonstrated in Supplementary Fig. 17. Remarkably, when the current is at 3 mA (Supplementary Fig. 16b), the discharging time is 146 s, revealing a large releasing current and long powering time of MSC. The charging/discharging curve at 0.1 mA/cm^2^ is also carried out and shown in Supplementary Fig. 18.

The results demonstrate that the as-fabricated MSCs possess a high bearable current to satisfy the current threshold of commercial electronics and drive high energy demanding appliances. In addition, the IMSCs display good electrochemical stability, exhibiting similar CV curve shapes as the increase of the cycling (Inset of Supplementary Fig. 16c). They remain 85% of the original capacitance after 3000 repeated cycles without any obvious declination (Supplementary Fig. 16c). The declination of capacitance is ascribed to the slight exfoliation of the graphite electrodes caused by the electrochemical intercalation reactions of [EMIM]^+^ into the graphite lattice during repeated charge and discharge cycles^31^. Coulombic efficiency is 90.7% after 3000 cycles comparing with the initial value of 92.0% (Supplementary Fig. 16d). ESR of IMSCs during the cycling process is also measured. As shown in Supplementary Fig. 16e, the slop of Nyquist plot at low frequency becomes smaller with the increase of cycle numbers, indicating the charging process controlled by the electric double layer mechanism gradually converts to ion diffusion limitation. This phenomenon may be explained by the intercalation of [EMIM]^+^ into the graphite electrodes causing inhomogeneous pore structure and roughness surface in electrode, thus slowing down the ion transport in the nonuniform pathway from the electrolyte to the electrode surface^31,32^. After enlarging the high-frequency area (Supplementary Fig. 16f), the ESR (the intercept of the *X*-axis) gradually increases from 19.8 to 29.8 Ω after 3000 cycles. It implies the interface resistance between the electrodes and electrolyte slightly increases^35^.

Moreover, energy density and power density are crucial factors to evaluate the performance of energy storage devices. As shown in Fig. 3e, the energy densities of IMSCs are from 273.1 μWh cm^−2^ to 463.1 μWh cm^−2^ and power densities are from 6.7 mW cm^−2^ to 2.0 mW cm^−2^ correspondingly. It is worth noting that the IMSCs display a supreme and the highest energy density among all planar MSCs including hybrid MSCs, even commercial thin-film batteries (350 μWh cm^−2^) (Fig. 3e)^25,36–61^. Besides, the Ragone plot of volumetric and gravimetric performance of IMSCs are also presented in Supplementary Fig. 19.

The self-discharge and leakage current data are shown in Supplementary Fig. 20. The self-discharge time is 2.3 h from 3 V to 1.5 V, indicating the stored energy can be kept for a certain time in this device compared with other MSCs and macro-supercapacitors (Supplementary Table 2). The leakage current of IMSCs is 0.2 mA after 12 h. The slightly high self-discharging rate may be caused by migration of the active electrolyte^62^ and the oxygen-containing functional groups of high-surface-area AC in the diffusion-controlled process^63,64^.

### Mechanism of high performance of IMSCs

The mechanism for IMSCs with the high energy storage capacity is proposed as following.

1. The structure design benefits the electrochemical performance. Comparing the planar IMSCs with conventional stacked AC-based SC (SAC-SC) under the same area of electrodes, the planar interdigital IMSCs present a higher electrical performance than SAC-SC. Since the EIS could reflect the electrochemical dynamic process of electrodes, we compared the EIS behaviors of these two types of devices (Supplementary Fig. 21a). It is shown that the IMSCs exhibit a lower ESR value of 19.8 Ω than that of SAC-SC (60.8 Ω), indicating a smaller internal resistance of the integrated microdevices. Moreover, the IMSCs display almost twice the enclosed area of CV curves than SAC-SC (Supplementary Fig. 21b), indicating superior electrochemical performance. The significant difference in the electrochemical performance could be mainly attributed to the different electrode structure designs. The integrated IMSCs consist of planar interdigital electrodes which provide a direct charge transfer path between electrodes, leading to a faster-exchanging rate of matters and charges than the SAC-SC with the separator between electrodes.
2. The wide voltage window in this work is another important factor to achieve high energy density. Since the electrolyte of [EMIM][TFSI] could afford a brilliant voltage of 3 V in this system, the energy density improves greatly based on the wide potential range.
3. To investigate the electrochemical storage mechanism, the capacitive and diffusion-controlled contributions of the current are performed in Supplementary Fig. 22. The result shows that a larger proportional capacitance is mainly contributed by electrostatic interactions between electrolyte ions and AC particles^29^. Besides, small parts of [EMIM]^+^ can also insert into graphite layers at 2.2 V and provide an extra pseudo-capacitance (Supplementary Fig. 23a)^32^. This can be reflected by a small redox peak in the CV curves under the fast charging and discharging process (Fig. 2b).
4. In traditional hybrid ion capacitors, the hybrid MSC usually contains one battery electrode to increase energy density and one supercapacitor electrode to improve power density, such as AC//graphite^65^. The energy density demonstrates the storage of charges while the power density is like the import/export gate of the charges. Owing to the cross-section of MSC electrodes consisted of two layers (AC layer and graphite layer), the two layers possess an equal chance to store/release charges during the energy storage procedure. As a result, at the moment of releasing/storing, AC with large surface area plays a major role presenting a quite large power density, but with time going on, graphite featured high insertion space consequently also participates in the energy storage process, therefore, increases the energy density (also shown in Supplementary Fig. 16e where the slope of low frequency gets lower showing the pseudo-capacitor behavior becomes evident). However, although the capacitance is mainly contributed by the AC layer, the capacitance is unsatisfied (36.5 mF cm^−2^) when replacing GP with aluminum foil under the same thickness of 50 μm (Supplementary Fig. 23b). It is assumed that the coordination of AC, GP, and [EMIM][TFSI] electrolyte leads to the large energy density and capacitance. Furthermore, a combination of AC and graphite in one electrode coincidentally balances the mass capacitance of two electrodes equally, which is regarded as the best proportional choice in a hybrid device.

### Optimization of an antenna of IWC-MSCs

As the antenna of IWC-MSCs, the charging coils enable MSCs with repetitive and sustainable charging capability without any additional electrical port. To optimize the collection of the electric energy from the magnetic field, we investigated the main parameters of WCC as a wireless charging part (Supplementary Fig. 24a), including a gap of neighboring coils, turns of coils, and the distance between the receiving antenna and the transmitter. As displayed in Supplementary Fig. 24b, we find that when the gap between adjacent coils is 0.8 mm, the receiving voltage is ~5.8 V, which is higher than the signals with the gaps of 1.0 and 1.2 mm. It indicates that a narrow gap of neighboring coils is more efficient in the output of signals. On the other way, the distance between antenna and signal transmitter is another important factor of large signal reception. With the distance increasing from 0.5 cm to 1.0 cm, the sensing voltage signal of the wireless charging part rises (Supplementary Fig. 24c). While it will fall back when continuing to increase the distance to 1.5 or 2.0 cm because of the changeable efficiency influenced by transmission distance. Besides, owing to the controllable laser ablation technique, the number of coil turns is also investigated. When increasing the turns from 5 to 7, the received current raises from 2.8 mA to 3.3 mA, indicating a higher charging rate and shorter charging time (Supplementary Fig. 24d). Considering that more turns mean a larger footprint of the device and 5 turns of the coil is enough for induction in this system, we adopt 5 turns of a coil as an antenna. Therefore, the optimal parameters of the WCC are 0.8 mm of the gap, 1.0 cm of the transmission distance, and 5 turns of the coil, leading to a high receiving current at 2.8 mA. In addition, the wireless charging part does not influence by the existence of the AC layer on WCC, showing the strong anti-interference ability (Supplementary Fig. 25). The inductance performance of the antenna is also characterized by the alternating current impedance method (Supplementary Fig. 26), which demonstrates good frequent stability of the charging coils as an antenna.

Corresponding alternative current and voltage in the transmitting coil of the transmitter are detected by a multimeter (Supplementary Fig. 27), which is 33.9 mA and 0.9 V, respectively. The transferring power efficiency of the wireless charging is 52.8%, indicating that the as-fabricated graphite WCC is a credible inductive antenna in this energy conversion system and the overall wireless charging system could be applied in practical applications.

### Testing performance of the integrated IWC-MSCs

**Fig. 4:**
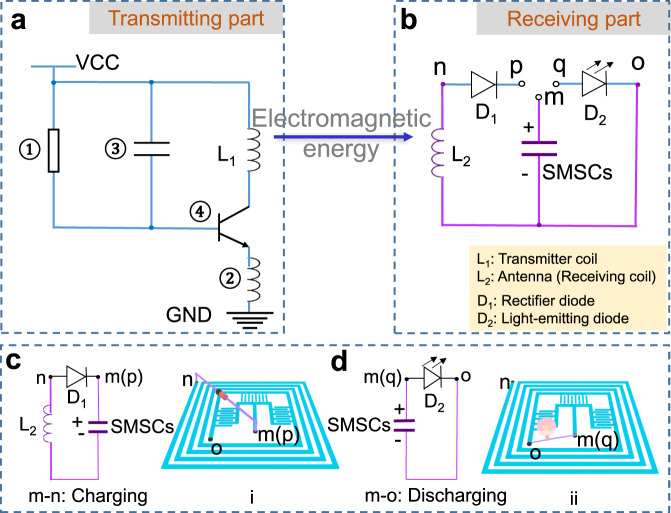
Circuit diagram and working mechanism of the wireless charging system. **a** Circuit diagram of wireless transmitting part and (**b**) receiving part. **c** Schematics of the circuit while connecting points “m” and “p” of IWC-SMSCs to charge SMSCs (i). **d** Schematics of the circuit while connecting point “m” and “q” of IWC-SMSCs to release the accumulated charges in SMSCs (ii), thus lighting a red LED. The IWC-SMSCs are in purple color in the circuit diagrams.

To evaluate the wireless charging performance of the integrated device, we have further investigated the charging and discharging capability of the IWC-MSCs. Generally, a wireless charging system consists of the transmitting part and receiving part as displayed in the circuit diagrams of Fig. 4a, b. Here, the transmitting part mainly relies on a square coil made from square-shaped copper wires (Supplementary Fig. 28a), which can be charged by a 10 V direct-current power source. For example, the IWC-SMSCs device is used as the receiving part (the purple lines in Fig. 4b), which is connected with an external LED bulb to detect the discharging signal (Supplementary Fig. 28b). The wireless charging mechanism follows the principles of *Electromagnetic Induction*, leading to the conversion of magnetic field energy to electrical energy. In the wireless charging process, the transmitting circuit delivers an alternating current in L_1_ (Fig. 4a) at first, causing a changeable magnetic field nearby. Then, the antenna (L_2_ in Fig. 4b) detects the magnetic field and induces alternating magnetic flux and current in receiving part. To achieve the stable charging process, the rectifier diode (D_1_ in Fig. 4b) is applied to rectify alternating current into direct current. When connecting “m” and “p” points (i), the wireless charging begins (Fig. 4c). Substantial charges produced from WCC are stored in the SMSCs of IWC-SMSCs. Details of energy receiving current and inductive charges of the IWC-SMSCs are shown in Supplementary Fig. 29. The sensed rectified current in the SMSCs gradually decreases (Supplementary Fig. 29a) and the induced electric charges steadily accumulate with the charging time going on (Supplementary Fig. 29b), indicating that the magnetic field energy is transformed into electric energy by WCC and the electricity is reserved in SMSCs. In contrast, when switch “p” to “q” points (ii), the discharging process begins (Fig. 4d). The charges stored in SMSCs are released and light the red-emitting diode (D_2_ in Fig. 4d). The entire wireless charging and detecting process is presented in Supplementary Movie 2.

### Application of IWC-IMSCs

**Fig. 5:**
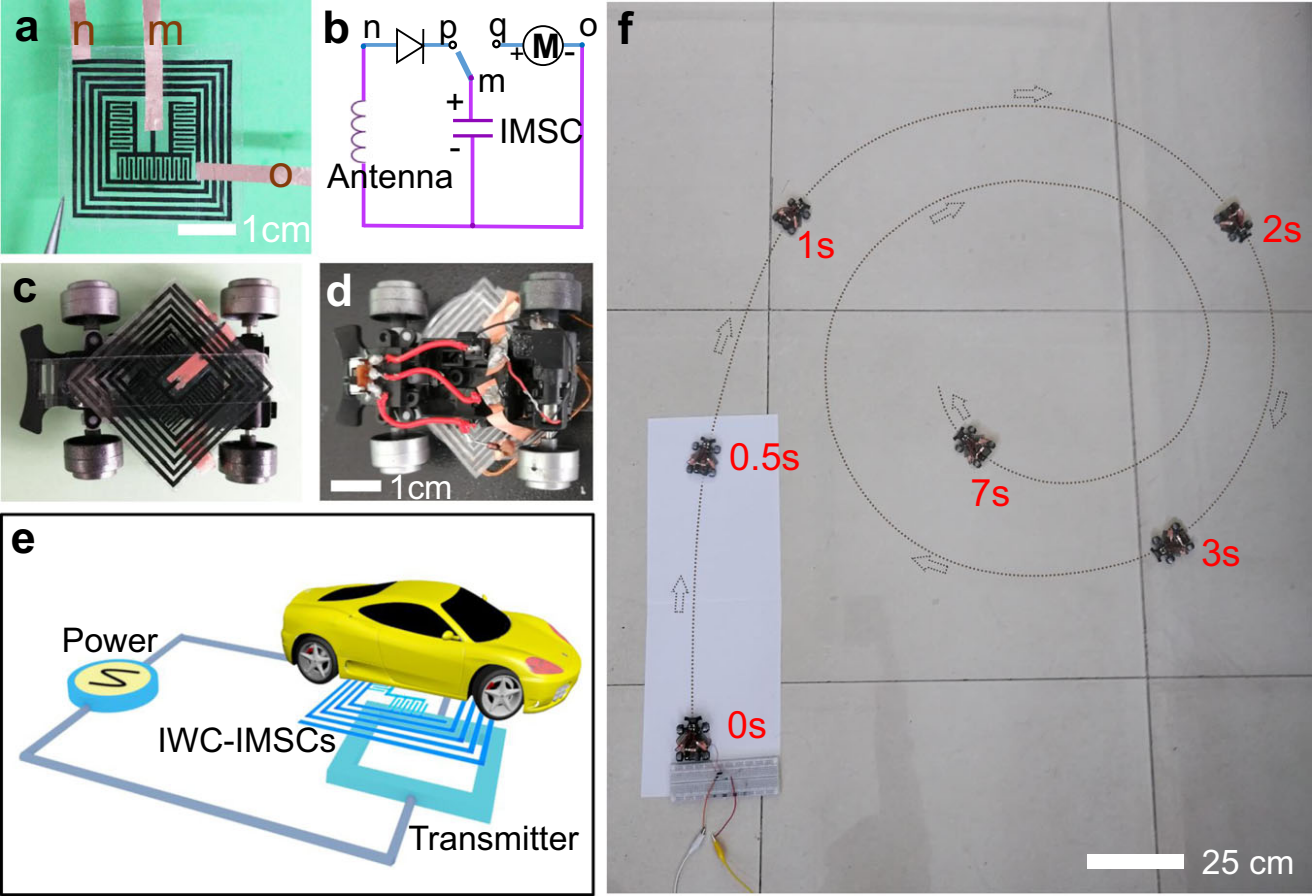
Assembling IMC-IMSCs with an electrical toy car as a power source. **a** An image of the connecting details of IWC-IMSCs using copper foils as conductors. “m”, “n”, “o” copper foils respectively correspond to “m”, “n”, “o” point in (**b**). **b** The circuit diagram of the IWC-IMSCs assembling with the car. **c** The downside of the car assembled with IWC-IMSCs. **d** The upside of the car connecting with a switch. **e** The schematic diagram of the wireless charging system. **f** The picture of the car with IWC-IMSCs running on the floor.

The high-performance and integration of the wireless charging MSCs endow them with the ability to be applied in practical mobile electronics. We find that an electrical toy car exhibits a starting current of 2.2 mA, which can be easily powered by the fabricated IMSCs directly replacing the commercial columnar battery (such as an AA battery). As present in Fig. 5a, b, copper foils are used as conducting wires to stick on the IWC-IMSCs, corresponding to m, n, o points. Like the structure of the circuit in Fig. 4b, the copper foil at the “n” point is connected with a rectifier diode, while the copper foil at the “o” point is assembled with an electro-motor of the car. The wireless charging and discharging process are controlled by using a switch (“m” point) in the circuit diagram. Figures 5c, d demonstrate the photographs of IWC-IMSCs on a commercial toy car (front and back). This integrated wireless charging energy storage device is easily attached to the exterior of the car without complex fixing accessories, indicating good environmental adaptability and operability. To complete the wireless charging process, the car with IWC-IMSCs is used as the wireless receiver and placed nearby the transmitter (Fig. 5e). When the switch is at the “p” point, the IWC-IMSCs of the car are charged by an antenna in a magnetic field. After wireless charging for 6 min, the induced electric charges by WCC reach ~312.5 mC. Meanwhile, the whole energy and power output of the IWC-IMSCs achieve 0.18 mWh and 45.9 mW, respectively, which can drive an electrical toy car immediately once switching to “q” point (Fig. 5f and Supplementary Movie 3). The energy transfer efficiency from the electrical energy received of the WCC to the output energy of IMSCs is calculated as 53.8% (shown in Supplementary Fig. 30).

In addition, the wireless charging time depends on the wireless charging transmitter (Fig. 4a), which can be shortened by increasing the charging current. Since the higher voltage of the power source in the transmitting part provides more transmitting energy to the transmitting coils, a high charging current could be achieved by increasing the applied voltage of the transmitting part. After that, the transmitting coils have mutual inductance with receiving coils (also named WCCs), and induce stronger signals in the receiving part through the electromagnetic field, thereby generating larger receiving current and voltage in WCCs to charge MSCs of IWC-MSCs. When the voltage increases from 10 V to 12 V, the charging current is enforced accordingly and the charging time can be shortened to 5 min (Supplementary Fig. 31). The charge is 310.3 mC, very close to the accumulated charges (312.5 mC) under 10 V of the power voltage (Supplementary Fig. 30a). However, owing to the limitation of the maximum withstand voltage of the equipment, the long-term work under high load voltage is easy to destroy the transmitter. Considering the voltage of 12 V has exceeded the voltage limit of the bearable voltage of the transmitting circuit, we choose 10 V as the transmitting power source and charge for 6 min. The integration of wireless charging into MSCs greatly simplifies the practical charging process of electronics and promotes important applications such as moveable charging.

## Discussion

In conclusion, we developed integrated and flexible wireless charging MSCs device by combining WCC and MSCs electrodes together through an ingeniously designed pattern, which is for the convenience of contactless charging and rapid energy storage. This design enables the IWC-MSCs with a seamless contact structure, and with high conductivity and corrosion resistance properties by virtue of the oneness graphite material. Moreover, the as-prepared IMSCs of IWC-IMSCs possess an ultrahigh areal capacitance (454.1 mF cm^−2^) and a record high energy density (463.1 μWh cm^−2^), superior to the reported planar MSCs. Furthermore, while assembling IWC-IMSCs with an electrical toy car, the car can be driven immediately and ran quickly after only 6 min wireless charging. This work not only inspires the designs of integrated electrical device fabrication but also advances the convenience of miniature and portable electronics.

## Methods

### Materials

Chemicals are from commercial sources with no further treatment. GP (~50 μm thickness) and AC specialized for supercapacitor are purchased from XFNANO Company of China. PVDF and NMP are analytical grades purchased from Alfa Aesar. Ketjen Black is used as a conductive block (CB) and bought from Dodochem company in Suzhou city of China. [EMIM][TFSI] is purchased from Innochem company in China. The toy car (6.5 × 5 × 4 cm^3^, mode number, 8024) is bought from Chengzhang toy company of Taobao shop in China.

#### Preparation of MSC

1. *Preparation of IWC-MSCs pattern*. In total, 0.8 g AC, 0.1 g PVDF, 0.1 g CB and 4 ml NMP were added together, where AC has functioned as activated materials for capacitance and PVDF acted as a binder. NMP was the solvent to dissolve all materials together. The mixture was ground for 20 min to obtain a homogeneous viscous slurry. Then, a planar bladder was used to coat the slurry on one side of GP to make sure the surface smooth. After drying the AC coated paper in a vacuum oven for 24 h at 60 °C, one AC layer coated GP was obtained. If there is further need, the other side of GP is coated with the same AC layer in the way. Double-layered GP was pressed by 5 MPa pressure to make the GP–AC paper tight. The specific mass of each AC layer on SMSCs and IMSCs were 1.7 ± 0.12 mg/cm^2^ and 3.4 ± 0.25 mg/cm^2^ respectively for each side. To obtain interdigital electrodes and WCC pattern, we used a laser machine (5 W) to scribe the as-prepared GP–AC paper. The total number of interdigital electrodes of SMSCs and IMSCs in IWC-MSCs were 24 and 39, respectively. Besides, the gap of WCC changed with the requirement (Supplementary Fig. 24b). After thorough ablation, the achieved pattern was transferred on PET tape (a kind of highly adhesive tape on one side), which was as the substrate of the device. The gap and the width of the interdigital electrodes in MSCs are all 400 μm.
2. *Assembly of IWC-MSCs*. The copper foil was cut into stripes functioning as the conductive wires in the IWC-MSCs and adhered with electrodes by conductive silver adhesives. To ensure no reaction related to the copper wire, each copper foil was well protected by PET tape to avoid contact with graphite and electrolyte.

For the encapsulation process, the patterned MSCs electrodes and coils fabricated by laser ablation were needed to be encapsulated twice with a high adhesive PET tape. The wireless charging graphite coils (excluding MSCs electrodes) were firstly covered with a piece of PET tape, and sealed by pressing it several times to avoid contact between graphite coils and electrolyte. Then, we carefully dropped the ionic liquid electrolyte [EMIM][TFSI] between the MSCs electrodes (excluding wireless charging graphite coils, WCCs), and placed another PET tape on the top of the entire device. After pressing the PET tape several times until no obvious bubbles observed inside of the PET tape, the encapsulation was completed. All encapsulation processes were carried out in the air. Other junctions of the switch, wires, and copper strips were soldered by melted tin.

### Electrochemical measurements

All electrochemical tests were performed in a two-electrode system in the air. CV galvanostatic charge-discharge tests, and electrochemical impedance spectroscopy (EIS) were carried out on a CHI 760E electrochemical workstation (CH Instruments Inc. Shanghai, China). The calculations of areal capacitance, energy density, and power density are followed by our previous papers^1^, where the area of SMSCs and IMSCs is 0.3072 cm^2^ (~=0.3 cm^2^) and 0.4992 cm^2^ (~=0.5 cm^2^), respectively.

The areal capacitance *C*_a_ was calculated as follows.

1 $${C}_{a}({\mathrm{F/{{cm}}}}^{2})=\frac{I\left(A\right)\times \triangle t(s)}{\triangle V\left(V\right)\times A({\mathrm{{cm}}}^{2})}$$

or

2 $${C}_{a}({\mathrm{F/{{cm}}}}^{2})=\frac{1}{\nu \times ({V}_{h}-{V}_{l})\times {\rm{A}}}\int _{{V}_{i}}^{{V}_{f}}I(V){dV}$$

where *C*_a_, *I*, *Δt*, *ΔV*, and *A* are the areal capacitance (F cm^−2^), charge/discharge current (A), discharge time (s), discharge voltage (V), and the area of the electrode (cm^2^), respectively. $\nu$ is the scanning rate of CV curves (V  s^−1^); ${V}_{h}$ and ${V}_{l}$ are the highest and lowest voltage of the potential window (V), respectively. *A* is the area of electrodes (cm^2^); $\int _{{V}_{i}}^{{V}_{f}}I(V){dV}$ is the integrated area of CV curves. The areal capacitance is 430 mF cm^−2^ at the scanning rate of 20 mV s^−1^.

Calculation of energy density and power density

3 $$E\left({\mathrm{Wh/cm}}^{2}\right) = \frac{I(A) * \int_{{{t}_{b}}}^{{{t}_{e}}} V(t) * dt}{A({\mathrm{cm}}^{2})*3600}$$

4 $$P\left({\mathrm{W/{{cm}}}}^{2}\right)=\frac{E\left({\mathrm{{Wh/}}{{cm}}}^{2}\right)}{\triangle t\left({\mathrm{s}}\right)}\times 3600$$

where *E* and *P* are the energy density (Wh cm^−2^) and power density (W cm^−2^), respectively. *t*_*b*_ and *t*_*e*_ are the beginning and ending times of discharging; *V*(*t*) is the device voltage;$\int _{{t}_{b}}^{{t}_{e}}V(t)* {dt}$ is the integrated area from the galvanostatic discharging curves.

The induced electric charges (*Q*) of WCC in IMSCs after wireless charging was calculated by

5 $$Q({mC})=\int _{{t}_{B}}^{{t}_{E}}I(t)* {dt}$$

where *t*_*B*_ and *t*_*E*_ are the beginning and ending time of wireless charging; $I(t)$ is the induced current by WCC at real-time; $\int _{{t}_{B}}^{{t}_{E}}I(t)* {dt}$ is the integrated area from induced current curves.

The EIS tests were carried out in the frequency range of 10^6^ to 0.1 Hz with 5 mV of amplitude. Receiving rectified current signals of an antenna is tested by Keithley 2612 system sourcemeter controlled by a LabView-based data acquisition system while the 1N4148 rectifier diode is to transverse alternative current into direct current.

### Characterization measurements

The SEM images were carried out on SUPRA 55. The laser ablation is carried out in the air at room temperature by the 355 nm ultraviolet laser cutting system (Beijing LAJAMIN LASER Co., LM-UVY-5S-Y). Pulse width, 1 μs. Pulse frequency, 30 kHz. Density value, ~0.125 J cm^−2^. Scanning rate, 500 mm/s. Coil resistance is tested by KDY-1 four-probe resistivity tester.

### Reporting summary

Further information on research design is available in the Nature Research Reporting Summary linked to this article.

## Supplementary information

Supplementary Information

Description of Additional Supplementary Files

Supplementary Movie 1

Supplementary Movie 2

Supplementary Movie 3

Reporting Summary

## Data Availability

The data that support the findings of this study are available from the corresponding author upon request.
