# Supplementary material for: A seamlessly integrated device of micro-supercapacitor and wireless charging with ultrahigh energy density and capacitance
Source: Nat Commun. 2021 May 11;12:2647. doi: 10.1038/s41467-021-22912-8 (PMC8113435; doi:10.1038/s41467-021-22912-8)
Supplement: Supplementary file 1 — Supplementary Information [file 41467_2021_22912_MOESM1_ESM.pdf]

Supplementary Information

A Seamlessly Integrated Device of Micro-supercapacitor and Wireless Charging with Ultrahigh Energy Density and Capacitance

Chang Gao[1], Jiancheng Huang[2], Yukun Xiao[1], Guoqiang Zhang[1], Chunlong Dai[1], Zengling Li[1], Yang Zhao[1*], Lan Jiang[3], Liangti Qu[1, 4*]

[1]Beijing Key Laboratory of Photoelectronic/Electrophotonic Conversion Materials, School of Chemistry and Chemical Engineering, Beijing Institute of Technology, Beijing 100081, P. R. China

[2]School of Microelectronics, Tianjin University, Tianjin 300072, P. R. China

[3]Laser Micro-/Nano-Fabrication Laboratory, School of Mechanical Engineering, Beijing Institute of Technology, Beijing 100081, P.R. China

[4]Key Laboratory of Organic Optoelectronics & Molecular Engineering of Ministry of Education, Department of Chemistry, Tsinghua University, Beijing 100084, P. R. China.

Corresponding author. E-mail address: yzhao@bit.edu.cn (Y. Zhao); lqu@mail.tsinghua.edu.cn (L. Qu)

Supplementary Note 1: Advantages of the materials, design and fabrication

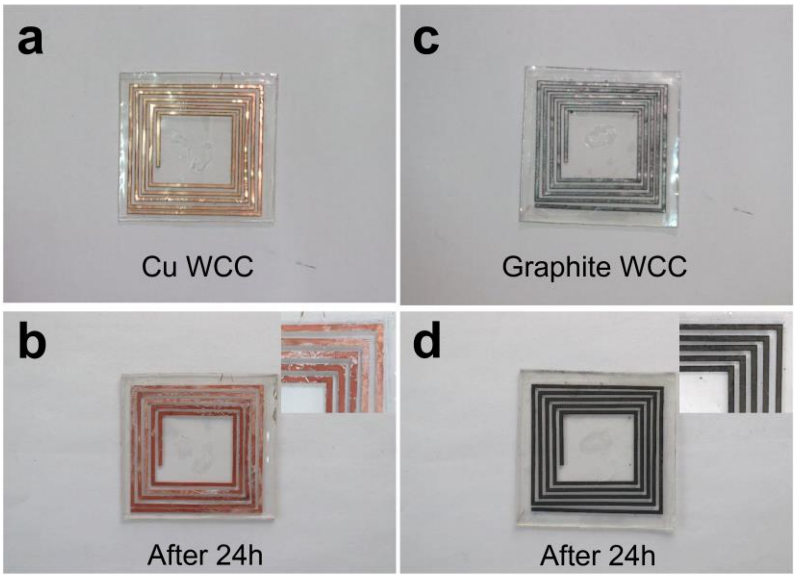

Supplementary Fig. 1 Optical pictures of WCC made by metal (copper) in a) initial state and b) placed in air for 24 hours after dipped in 1M H_2SO_4 for 5s. The inset is the magnified picture of copper coil; The WCC made by graphite in c) initial state and d) placed in air for 24 hours after dipped in 1M H_2SO_4 for 5s. The inset is the magnified picture of graphite coil. Obviously, the copper WCC is corroded rapidly and the graphite WCC exhibits no obvious change, showing the outstanding corrosion resistance of graphite.

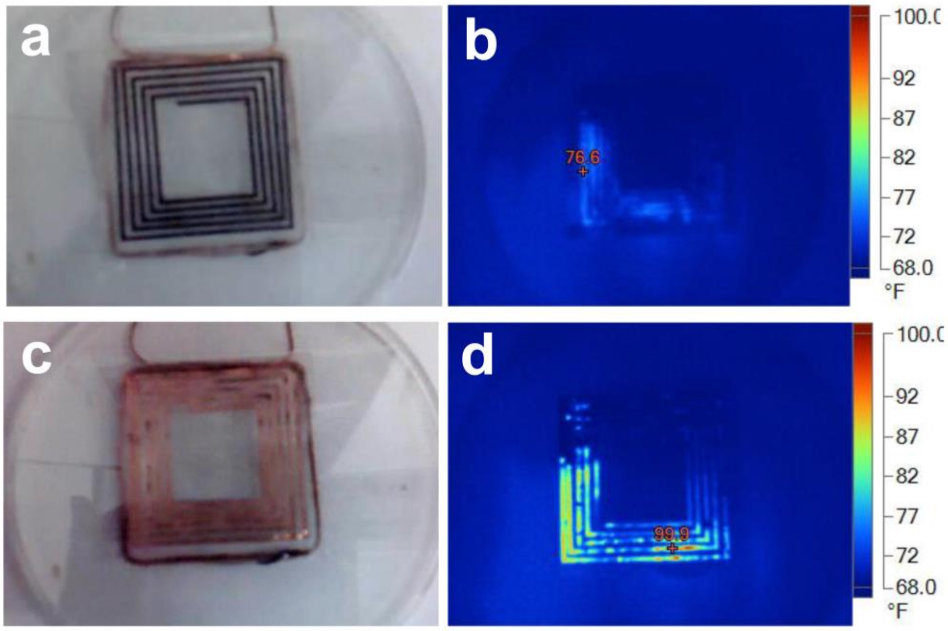

Supplementary Fig. 2 Infrared images of the temperature variation recording of a, b) graphite WCC and c, d) copper WCC during wireless charging. After charging for 6 minutes, the graphite WCC is 76.6°F of the temperature, much colder than the copper WCC (99.9°F), indicating lower heat generation than metal-based coil.

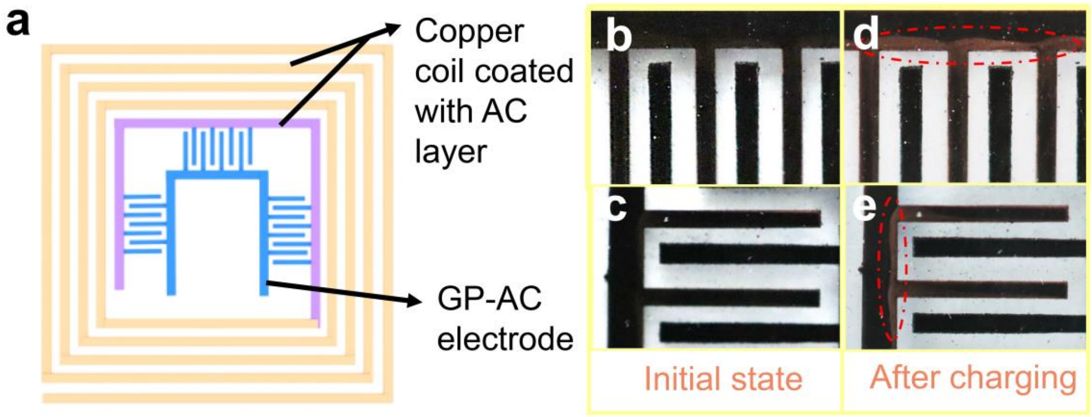

Supplementary Fig. 3 a) Scheme of the IWC-MSCs using copper as the antenna and one electrode of MSCs, the rest part is made of GP-AC electrode. b, c) Electrodes of IWC-MSCs with copper in initial state. d, e) Electrodes of MSCs with copper after repeated charging for 300 cycles under the scanning

rate at 40 mV/s. Red dotted circles demonstrate brown color change of copper coil as one electrode of MSCs. Besides, the test is very unstable when using copper as one electrode of IWC-MSCs at the initial state. In this case, graphite paper displays a superior stability as an antenna.

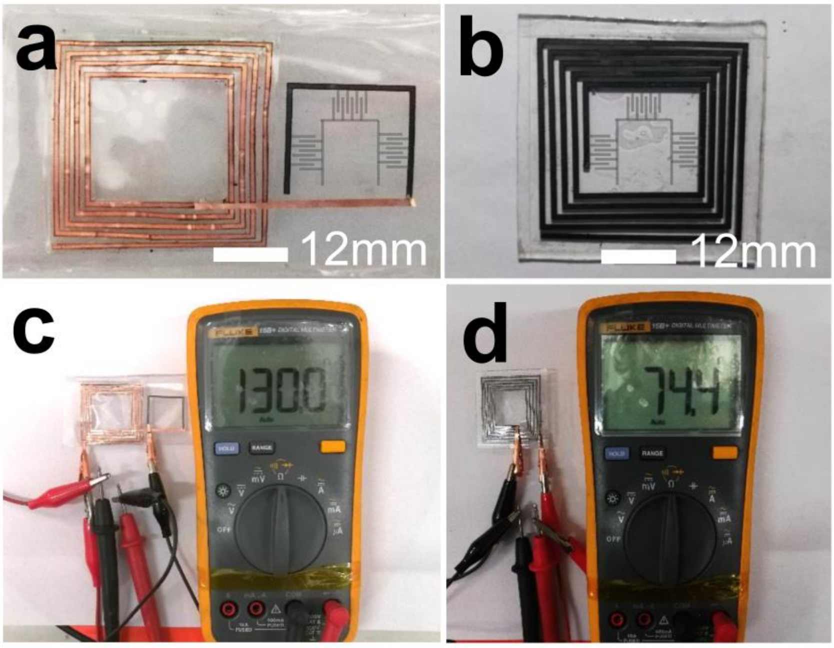

Supplementary Fig. 4 a) The optical picture of connecting copper-based WCC and GP-AC wire by copper foil using conductive silver adhesive. b) The optical picture of GP-AC WCC in our integrated device. The electrochemical resistance of c) the copper-based assembled device is 55.6 Ω higher than d) the integrated device. It could be concluded that the pattern design of IWC-MSCs produce lower electrical resistance than the stiffly integrating metal-based WCC with MSCs.

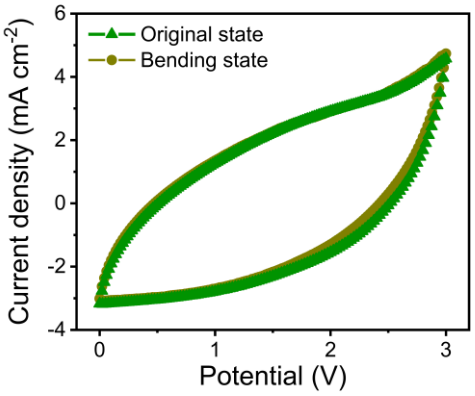

Supplementary Fig. 5 CV curves of a single MSC unit under bending at the angle of 90° state and original state.

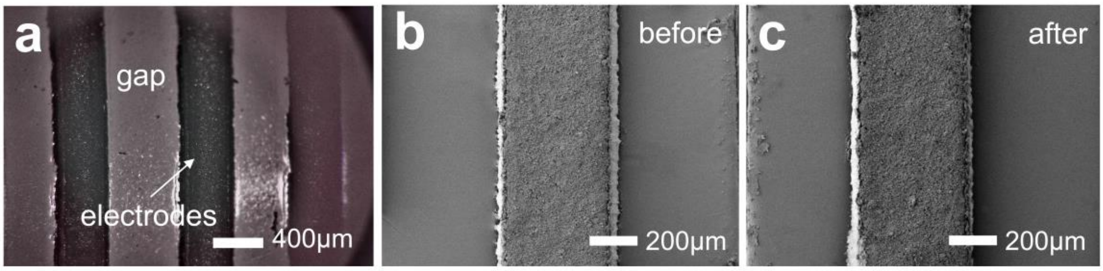

Supplementary Fig. 6 a) The optical image of the MSCs electrodes. b) SEM images of interdigital electrodes before bending and c) after bending for 90°. There is no evident crack in both the electrode and coil, demonstrating good flexibility of the device.

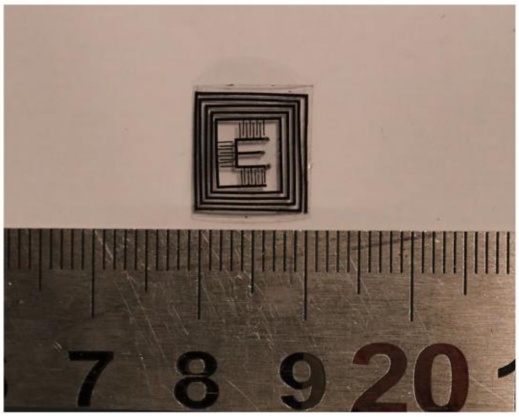

Supplementary Fig. 7 The optical image of the IWC-MSCs with an area about 1cm^2 fabricated by laser machine.

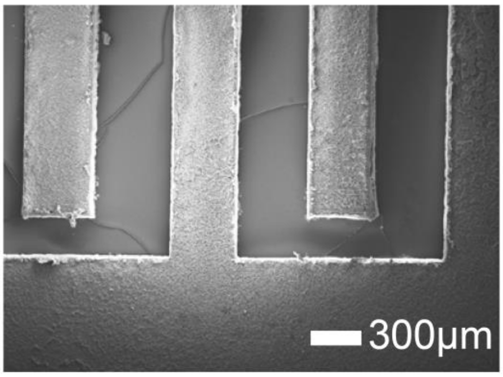

Supplementary Fig. 8 The SEM image of the interdigital electrodes of the MSCs in IWC-MSCs.

Supplementary Note 2: Calculation of IR_{drop} of the MSC

The IR_{drop} is calculated from the following equations.[s1,s2]

$$IR_{drop} = a + bI \qquad \text{(Equation S1)}$$

where a is the difference between the applied and real voltage of MSC (intercept of y axis in Supplementary Fig. 9a); b is the twice of the ESR of MSC (Supplementary Fig. 9a). Thus, the IR_{drop} is 0.17 V under the current density at 2 mA cm^{-2}, thus equations for the percentage of IR_{drop} (η, 5.5%) are:

$$\eta = \frac{IR_{drop}}{V_p} * 100\% \qquad \text{(Equation S2)}$$

$$I = S * I_d \qquad \text{(Equation S3)}$$

where I, I_d, S are the current, current density (2.0 mA cm^{-2}) and electrodes area (0.1 cm^2), respectively; V_p is the range of potential window (3V).

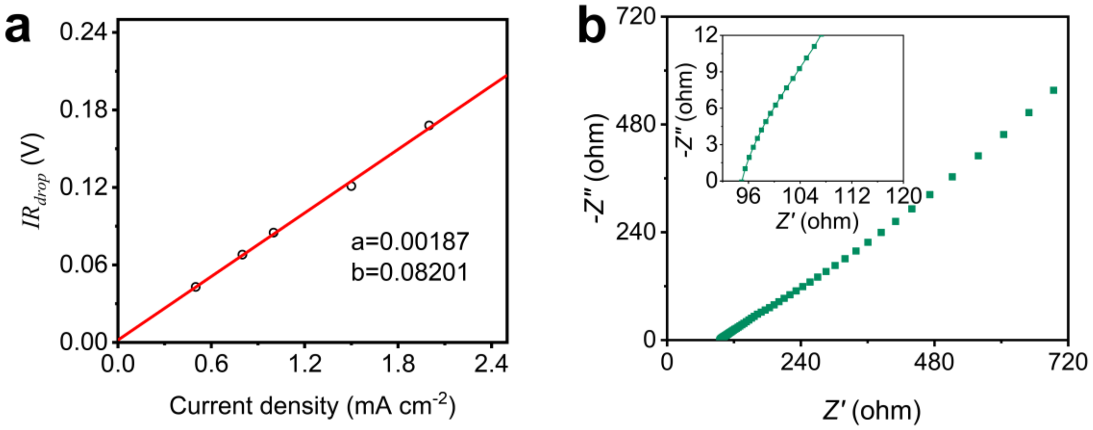

Supplementary Fig. 9 a) IR_{drop} related to MSC internal resistance *vs* different discharge current densities. b) The Nyquist plot of a single MSC unit; The inset is magnified details of the Nyquist plot.

Supplementary Note 3: Optimization of the device

Three different thicknesses of AC layers were prepared on GP substrate, in which both two sides of AC layers were 30±2 μm (Supplementary Fig. 11a, b), 50±2 μm (Supplementary Fig. 11d, e) and 60±2 μm (Supplementary Fig. 11g, h), respectively. By comparing their surface topography(Supplementary Fig. 11b, e, h) and electrochemical CV curves (Supplementary Fig. 12), we found that the electrochemical performance of the MSCs increased with the increase of thickness of AC layers at beginning from 30 to 50 μm, but then decreased slightly as the thickness increased to 60 μm. This can be attributed to that the thicker AC layers would prolong the distance of mass transfer and decrease the transfer rate. In addition, different thicknesses of the AC layers would also affect the laser processing process. In order to achieve clear and consistent electrode boundary (Supplementary Fig. 11c, f, i), thicker AC layers

need longer laser ablation time or higher laser power. According to above, 50 μm thickness of AC layer is selected appropriately.

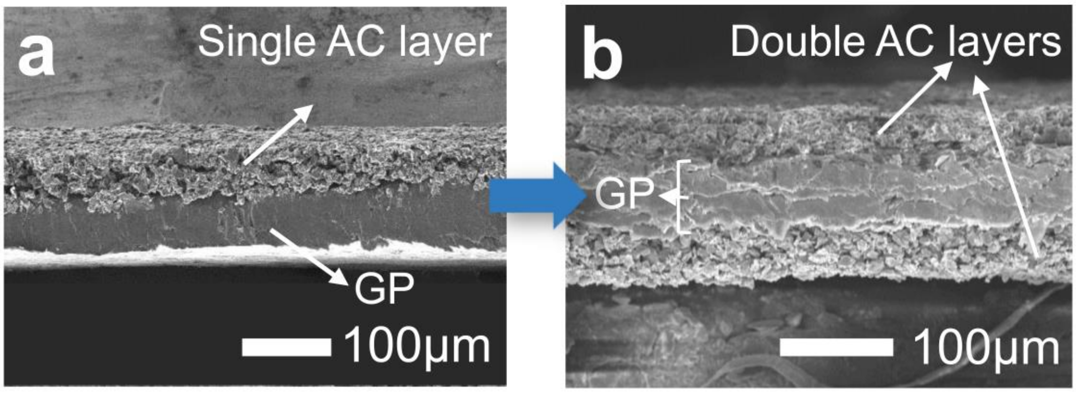

Supplementary Fig. 10 SEM image of the cross-section view of the a) SMSCs and b) DMSCs.

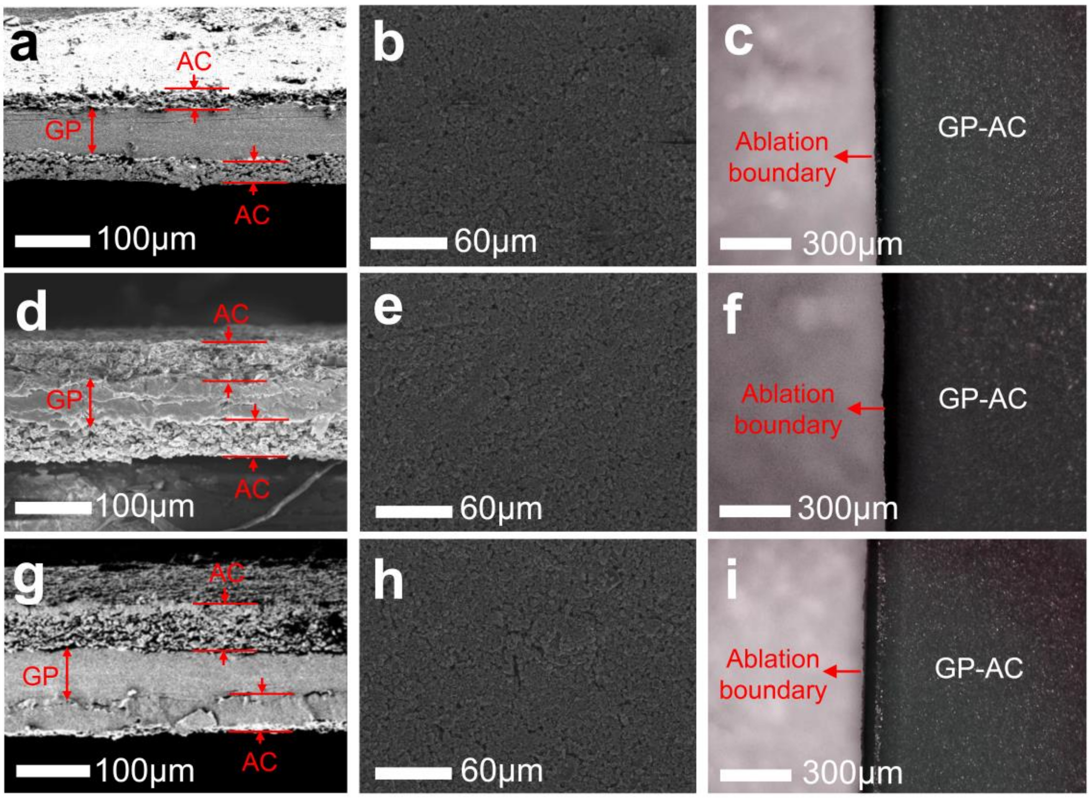

Supplementary Fig. 11 SEM images and microscopic images of the GP-AC with AC layer thickness at (a-c) ~30 μm, (d-f) ~50 μm, (g-i) ~60 μm, respectively. a, d, g) Cross-section images of 30 μm-thick, 50 μm-thick, and 60 μm-thick AC

layers on both sides of GP substrate, respectively. b, e, h) SEM images of the surface of 30 μm-thick AC layer, 50 μm-thick AC layer, and 60 μm-thick AC layer of GP-AC, respectively. c, f, i) The optical images of 30 μm-thick AC layer edge, 50 μm-thick AC layer edge, and 60 μm-thick AC layer edge after laser ablating for ~1s per centimeter, ~2s per centimeter, and ~3s per centimeter, respectively.

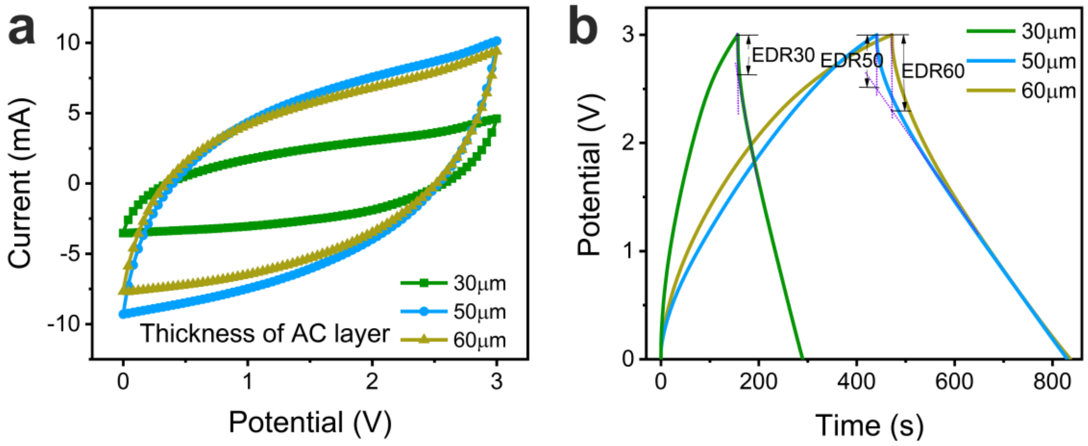

Supplementary Fig. 12 a) Comparison of CV curves of the GP-AC at the scanning rate of 100mV/s and b) Comparison of the equivalent distributed resistance (EDR) of galvanostatic charging/discharging curves for the GP-AC at 1mA when the thicknesses of AC layer are 30, 50, 60 μm per side, respectively. The EDR increased with the increase of activated carbon layer thickness accordingly due to the growing pore length of electrodes.[s3]

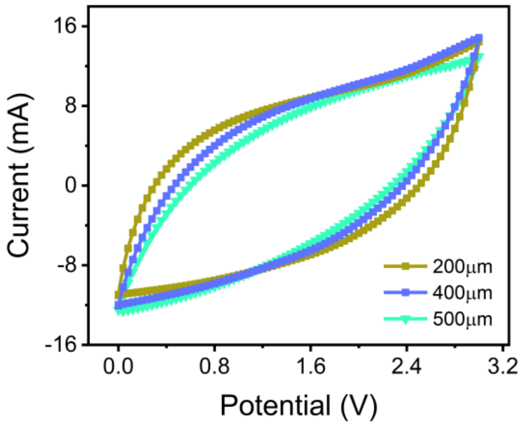

Supplementary Fig. 13 Comparison of CV curves at the scanning rate of 100mV/s when the gap between two adjacent electrodes are 200, 400, and 500 μm, respectively. The picture shows that with the electrodes gap increasing, the area of CV curves improves, so the 200 μm-gap MSCs presents the best capacitance. However, considering the operability of the device (too narrow gap of two electrodes is easy to connect), we choose 400 μm of the gap.

Supplementary Note 4: Characterization of the encapsulation of the device

After encapsulation with PET tape and standing 20 days in the air ambient conditions, the MSC encapsulated with PET tape exhibited a similar and stable electrochemical behavior with the initial one, showing good air stability (Supplementary Fig. 14). Besides, the unencapsulated MSC displays an inferior capacitance compared to encapsulated MSC. As demonstrated in Fig. 15a, the electrochemical CV curve of the unencapsulated MSC shows a smaller scanning area than the encapsulated one in the initial stage. Its capacitance decreases rapidly in the air (Supplementary Fig. 15b) and the capacitance

maintains only 82.2% after 1000 cycles under 40 mV/s scanning rate. It is considered that the rapid declination is due to the water and oxygen in the air that may react with $[EMIM]^+$, thus consuming parts of electrolyte ions and decrease the capacitance of MSC.[s4]

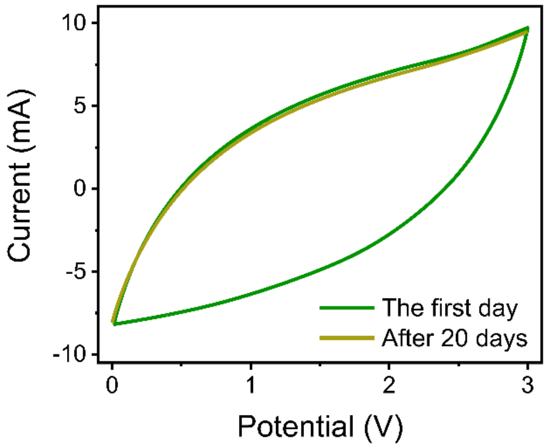

Supplementary Fig. 14 Comparison of CV curves of the encapsulated IMSCs before and after standing 20 days in the air ambient conditions. The scanning rate is 100mV/s.

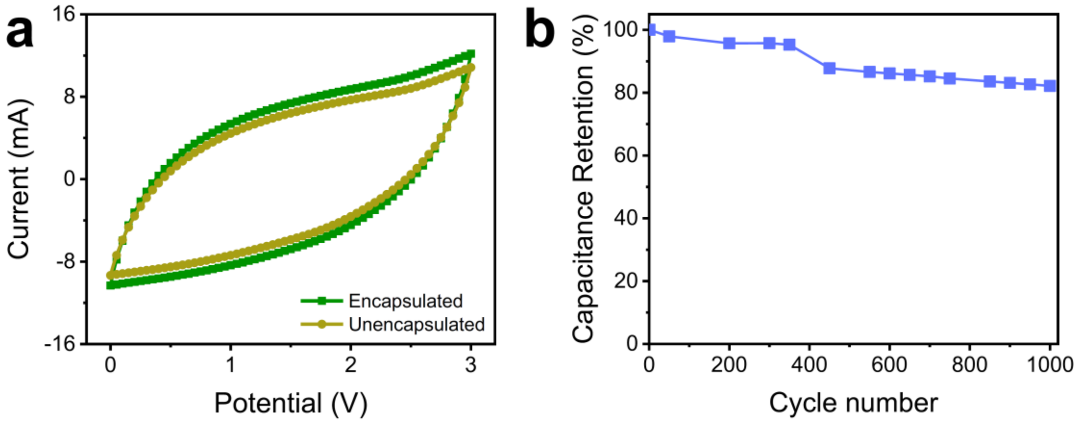

Supplementary Fig. 15 a) Comparison of CV curves of the encapsulated and unencapsulated IMSCs at the scanning rate of 100mV/s. b) The cyclic stability of IMSCs without encapsulation by PET tape.

Supplementary Note 5: Characterization of electrical performance of the optimized MSC

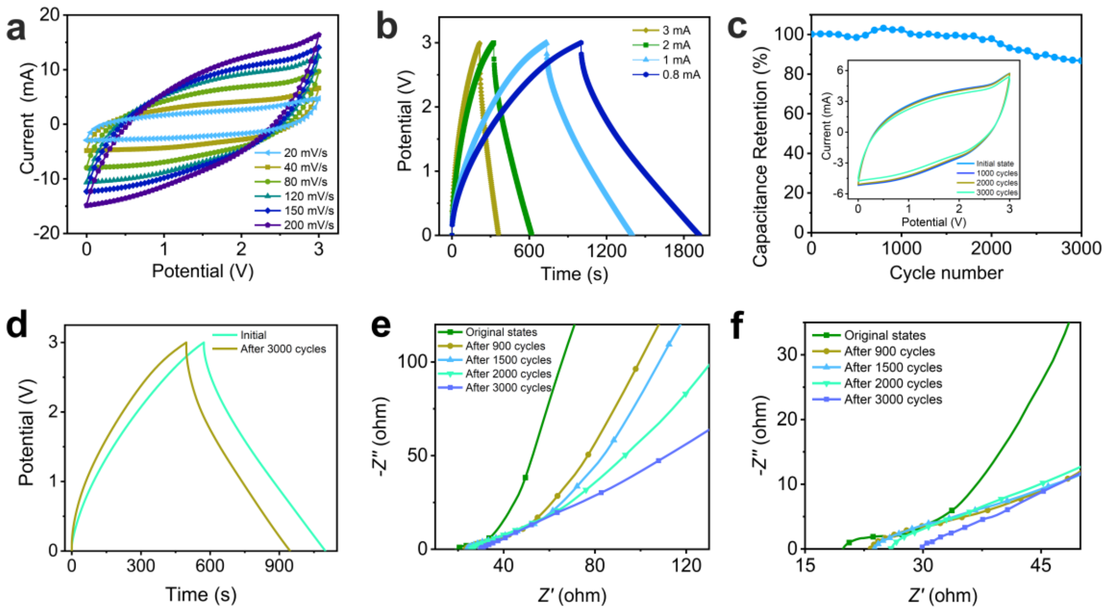

Supplementary Fig. 16 Electrochemical performance of the overall IMSCs. a) CV curves measured at scan rates of 20-200 mV/s. b) The galvanostatic charging/discharging curves of IMSCs under different current values. c) Cycling performance of IMSCs under scanning rate of 40 mV/s for 3000 repeated charging/discharging cycles. The inset is the CV curves during cycles. d) The galvanostatic charging/discharging curves of IMSCs at the current of 1 mA before and after 3000 cycles. e) The Nyquist plots and f) magnified Nyquist plots of IMSCs during repeated charging/discharging cycles.

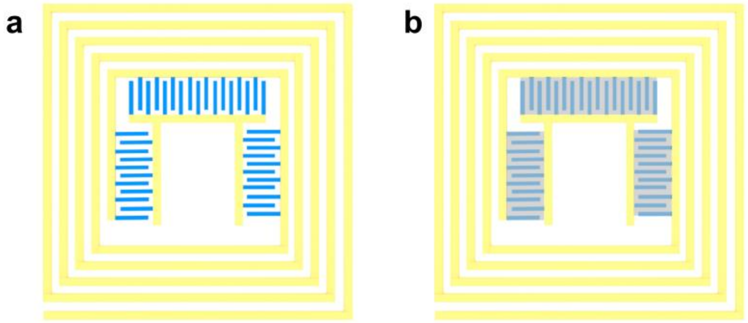

Supplementary Fig. 17 Diagrams of the calculation areas of a) electrodes area (the electrodes area is 0.50 cm^2 in blue color, and the corresponding areal capacitance is 454.1 mF cm^{-2}), and b) electrodes including the gap (the electrodes area is 1.32 cm^2 in gray color, and the corresponding areal capacitance is 171.7 mF cm^{-2}).

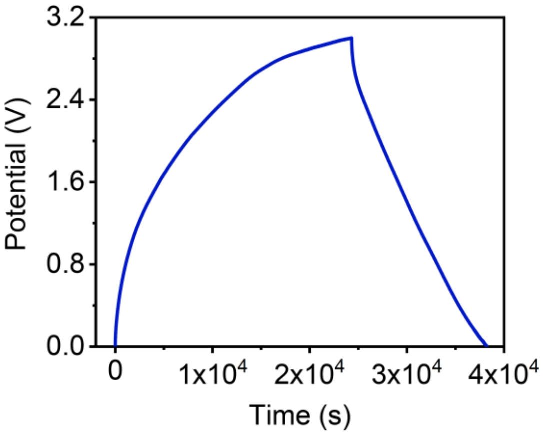

Supplementary Fig. 18 The galvanostatic charging/discharging curves of IMSCs under the current density of 0.1 mA cm^{-2}.

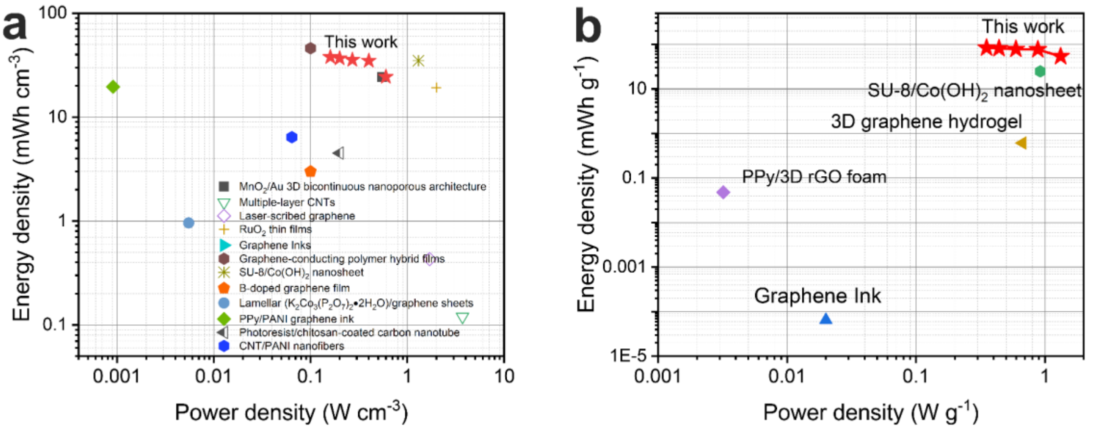

Supplementary Fig. 19 Ragone plots of a) volumetric and b) gravimetric performance of the IMSCs.[s5-s18] The energy density and power density of volume are 24.4~37.8 mWh cm^{-3} and 0.16~0.6W cm^{-3}, respectively, which are comparable to those well-developed planar MSCs reported previously. Besides, the gravimetric energy density is range from 53.7 to 83.3 mWh g^{-1}, and the gravimetric power density is from 0.35 to 1.32 W g^{-1}, superior to the state-of-art micro-supercapacitors.

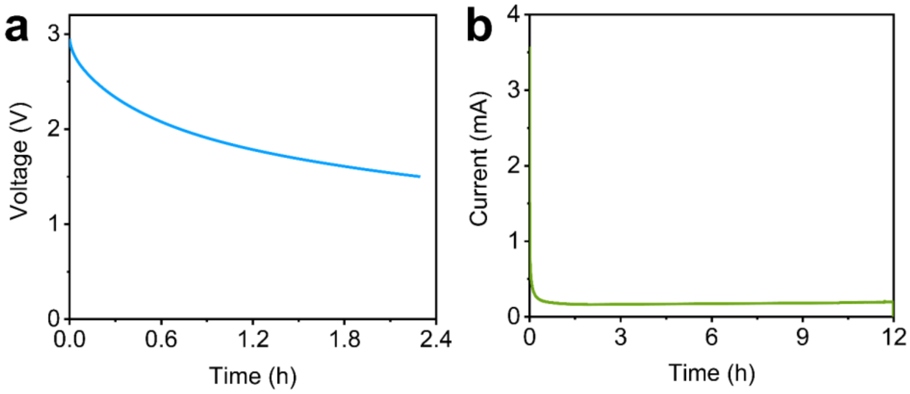

Supplementary Fig. 20 a) Self-discharge profile of IMSCs obtained immediately after charging to 3V. The voltage drops from 3 V to 1.5 V after 2.3 hours of self-discharging. b) Leakage current curve of IMSCs under the voltage of 3 V.

Supplementary Note 6: Mechanisms of the high performance of IMSCs

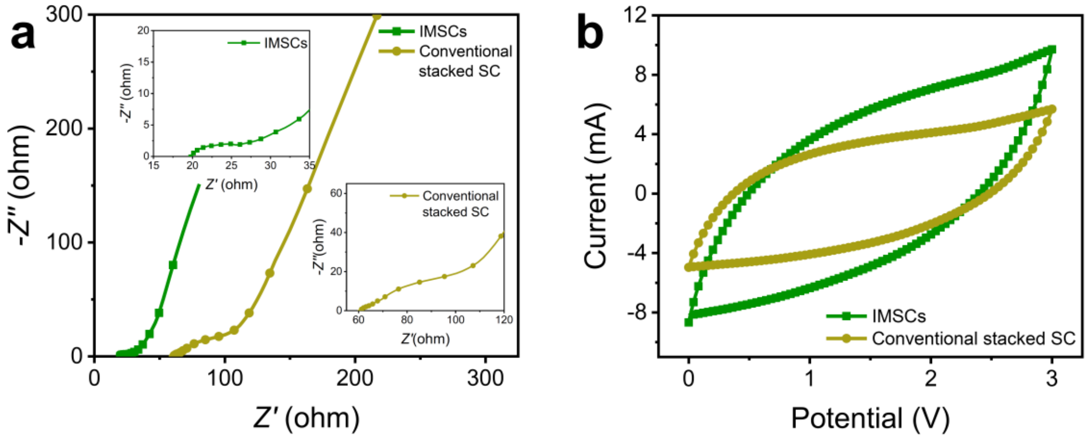

Supplementary Fig. 21 a) The Nyquist plot of IMSCs and conventional stacked SC (GP-AC electrodes have the same area with IMSC electrodes, and the filter paper is used as separator); The inset is magnified details. b) CV curves of IMSCs and conventional stacked SC measured at scan rates of 100 mV/s.

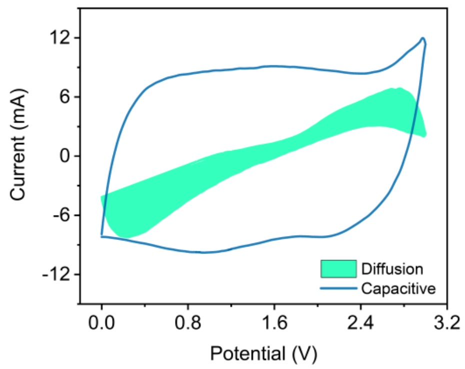

Supplementary Fig. 22 Contribution ratio of the capacitive-controlled charge under the scanning rate at 150 mV/s. This graph is calculated by $i = k_1 v + k_2 v^{\frac{1}{2}}$ and $i = av^b$, where $k_1 v$ and $k_2 v^{\frac{1}{2}}$ are capacitive and diffusion-controlled contributions of the current, and i is the current, v is the scanning rate, a and b are adjustable parameters.[s19]

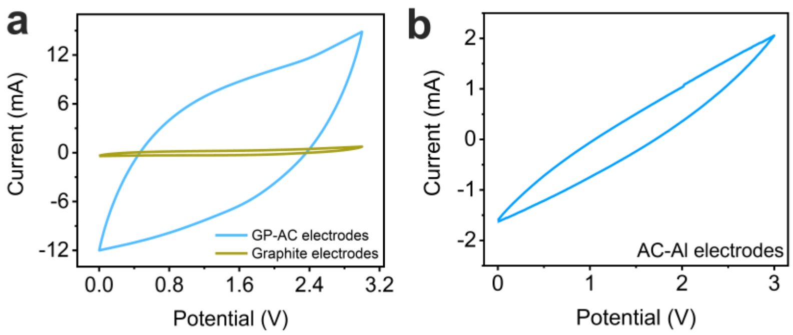

Supplementary Fig. 23 a) CV curves of IMSCs made of GP-AC electrodes and only graphite paper as the electrodes under 100 mV/s scanning rate. The enclosed area of CV curves of graphite electrodes presents the graphite also contributes a part of capacitance to IMSCs. b) CV curves of IMSCs made of AC coated aluminum electrodes under 100 mV/s scanning rate.

Supplementary Note 7: Optimization of wireless charging coil

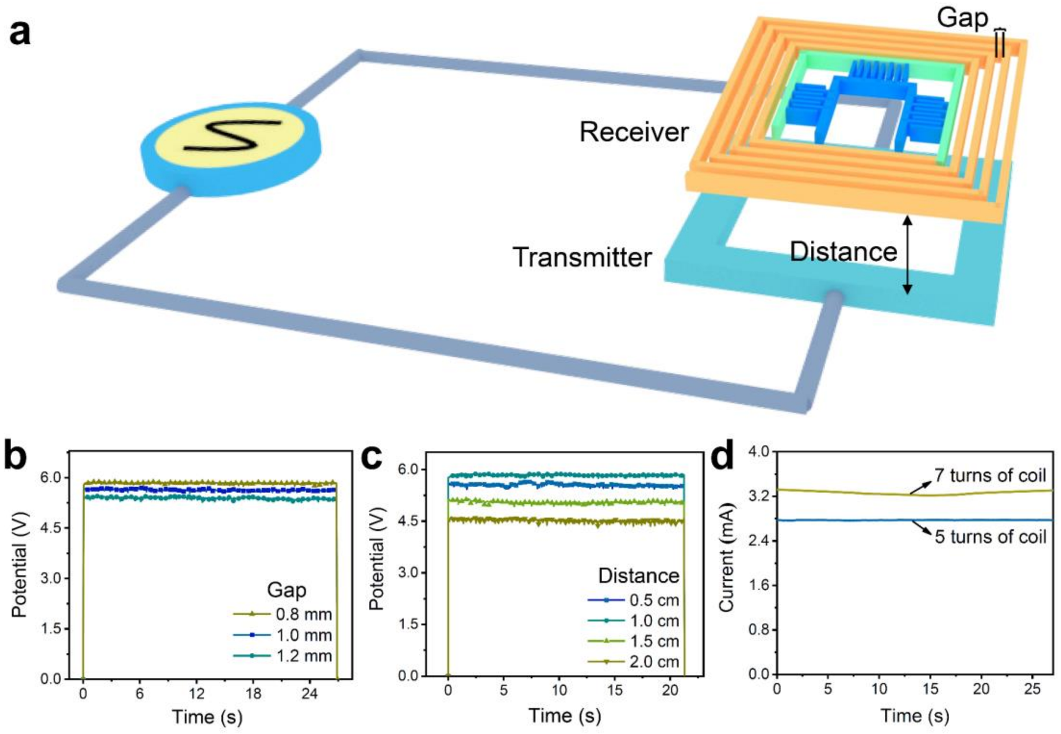

Supplementary Fig. 24 Parameters investigation of WCC in IWC-MSCs. a) A schematic of the WCC (orange color) in IWC-MSCs. In fact, the whole IWC-MSCs functioning as a receiver. b) Sensing voltage signals of the WCC with various gap and c) the distance between the WCC and transmitter. d) Sensing current signals of WCC with 5 or 7 turns of coil under the optimal state (0.8 mm gap and 1.0 cm distance).

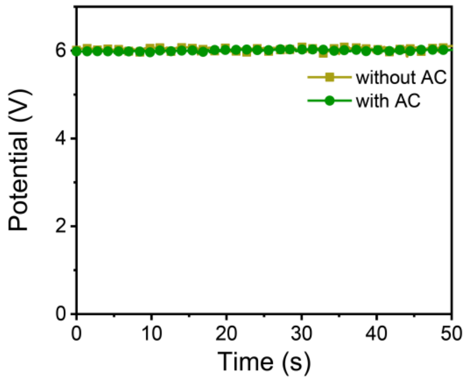

Supplementary Fig. 25 Receiving potential signals of the optimal WCC covered with AC layer and without AC layer placed in magnetic field.

Supplementary Note 8: Inductance performance of wireless charging coil

The frequency of the wireless charging coil was measured from 1 kHz to 600 kHz, and the real impedance, phase, imaginary impedance and inductance were obtained in Supplementary Fig. 26a-26c, respectively. In Supplementary Fig. 26a-26c, the real impedance within the whole frequency band has a small variation range, demonstrating the coil resistance (71.1Ω) and direct current loss are stable within the measurement range without causing fluctuations in

transmission efficiency. It is because the antenna is made of graphite, whose conductivity is based on π electrons between layers contributing less frequency affection. Compared with graphite WCC, the commercial antenna are usually made of metal, whose free electrons has a skin effect. When the frequency gets higher, the free electrons tend to accumulate together resulting in lower conductivity. The inductance of the WCC in Supplementary Fig. 26c was obtained according to the imaginary impedance shown inset by using the formula:

$$L(H) = \frac{Z''(\Omega)}{2\pi\mathrm{f(Hz)}} \quad \text{(Equation S4)}$$

where L is the inductance of the WCC; Z'' is the imaginary impedance of the WCC; f is the measured frequency.

Besides, by comparing Supplementary Fig. 26b and Supplementary Fig. 26c, both of the phase and inductance have experienced a steep decline near 140 kHz, so it can be inferred that the first resonant frequency of the coil is 140 kHz.

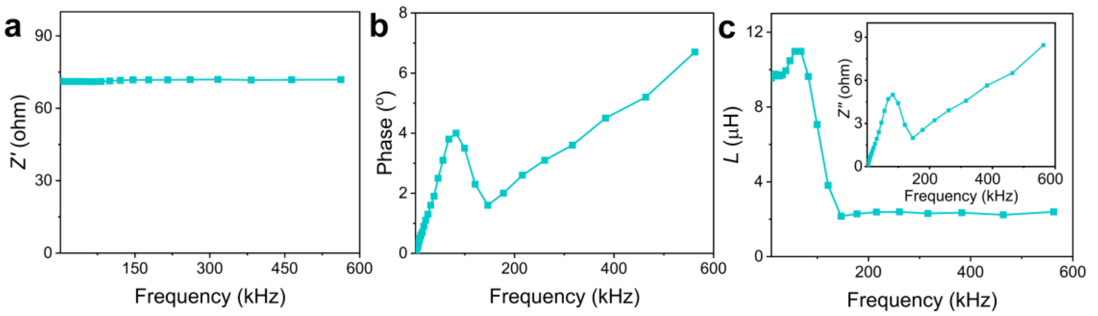

Supplementary Fig. 26 The inductance performance of WCC. a) Real impedance. b) Phase. c) Calculated inductance and imaginary impedance

(inset). L represents inductance of the coil.

Supplementary Note 9: Calculation of transmitting efficiency

The transmitting efficiency is calculated as following:

$$\eta = \frac{E_r}{E_t} * 100\% \quad \text{(Equation S5)}$$

$$E = P * t \quad \text{(Equation S6)}$$

$$P = U * I \quad \text{(Equation S7)}$$

where the E_r and E_t is the received and transmitted energy, respectively; P is the electrical power; t is the wireless charging time; U is the voltage and I is the current in Supplementary Fig. 27. The receiving current and voltage are the optimum values (2.8mA; 5.8V) of the WCC in Supplementary Fig. 24. In consequence, the transmitting efficiency of the wireless charging system is 52.8%.

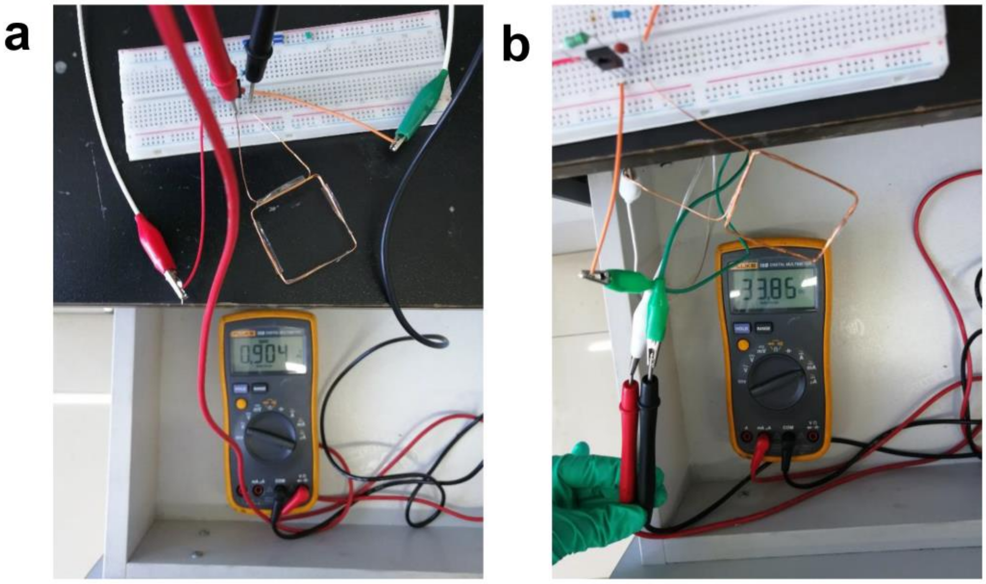

Supplementary Fig. 27 a) The alternative voltage and b) current of the transmitting coil of transmitter, which is 0.9V and 33.8 mA respectively.

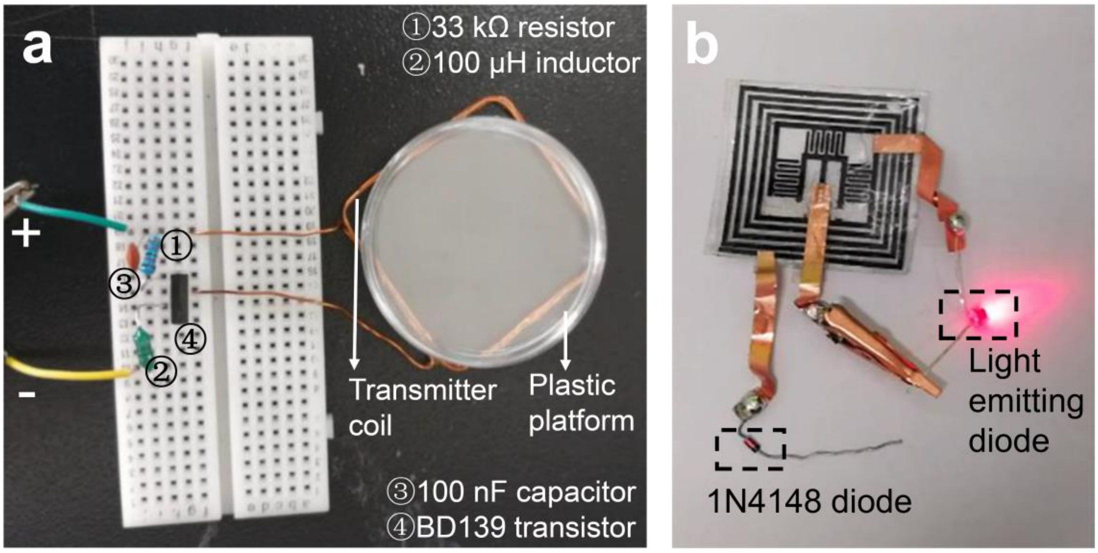

Supplementary Fig. 28 a) The transmitter system with a one-centimeter-high plastic platform (sometimes can remove away). The main transmitter coil is a square coil made by square-shaped copper wire. This transmitting system is charged by 10 V direct-current power source. b) The image of a red LED lit by SMSC of IWC-SMSCs after wireless charging for 2 minutes. The circuit diagram are consulted to the website (https://www.instructables.com/id/Easy-Wireless-LEDs) and references.[s20]

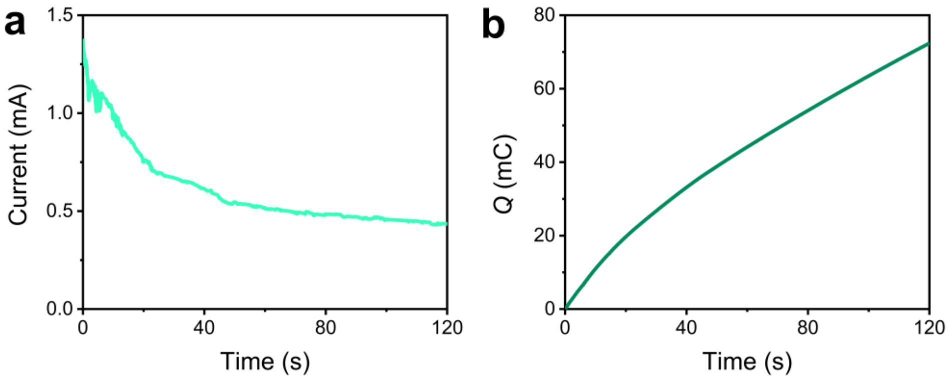

Supplementary Fig. 29 a) The received rectified current in SMSCs under wireless charging and b) the corresponding quantity of electric charges accumulated in SMSCs.

Supplementary Note 10: Calculation of the efficiency of energy transformation from induced electric energy in WCC to the output electricity of IMSCs

The discharging power of IMSCs and induced power of WCC are obtained from

$$P = V * I \qquad \text{(Equation S8)}$$

where V and I are the voltage and current of IMSCs or the WCC, respectively.

During wireless charging, the induced current of WCC after rectified is 2.7 mA and gradually drops to 0.43 mA after 6 minutes (Supplementary Fig. 30a). Meanwhile, the induced voltage of WCC increases from 2.5 to 4.6 V, showing the growing resistance of the circuit (slop of the curve in Supplementary Fig. 30b). The overall tendency of the induced power of WCC is downward because of sharply decreased current (Supplementary Fig. 30c).

After wireless charging for IMSCs, the output current and voltage are measured. The discharging current of IMSCs is large in Supplementary Fig. 30d, which reaches up to 17.4 mA, fairly enough for electrical appliance. However, the current deduces quickly due to the fast discharging nature of supercapacitor. After 400s discharging, the current is below 0.17 mA approaching to zero, thereby we ignore further discharging procedure. The corresponding discharging voltage decreases slowly from 2.73 to 2.53 V after 400 seconds (Supplementary Fig. 30e). Thus, the output power of IMSCs

achieves to 45.9 mW at the very beginning, providing splendid driving force to start the car (Supplementary Fig. 30f).

In total, the efficiency of energy transformation (from induced electric energy in WCC to the output electricity of IMSCs) is 53.8%. This efficiency (η) is calculated by Equation S9 and S10.

$$\eta = \frac{E_o}{E_i} * 100\% \quad \text{(Equation S9)}$$

$$E = \int_{t_b}^{t_e} P(t)dt \quad \text{(Equation S10)}$$

where E_o and E_i are the output energy of IMSCs and induced energy of WCC, respectively; t_b is the beginning time of the discharge of IMSCs or wireless charging of WCC; t_e is the ending time of the discharge of IMSCs or wireless charging of WCC. $\int_{t_b}^{t_e} P(t)dt$ is the integral area of Power-Time curves of Supplementary Fig. 30c or f.

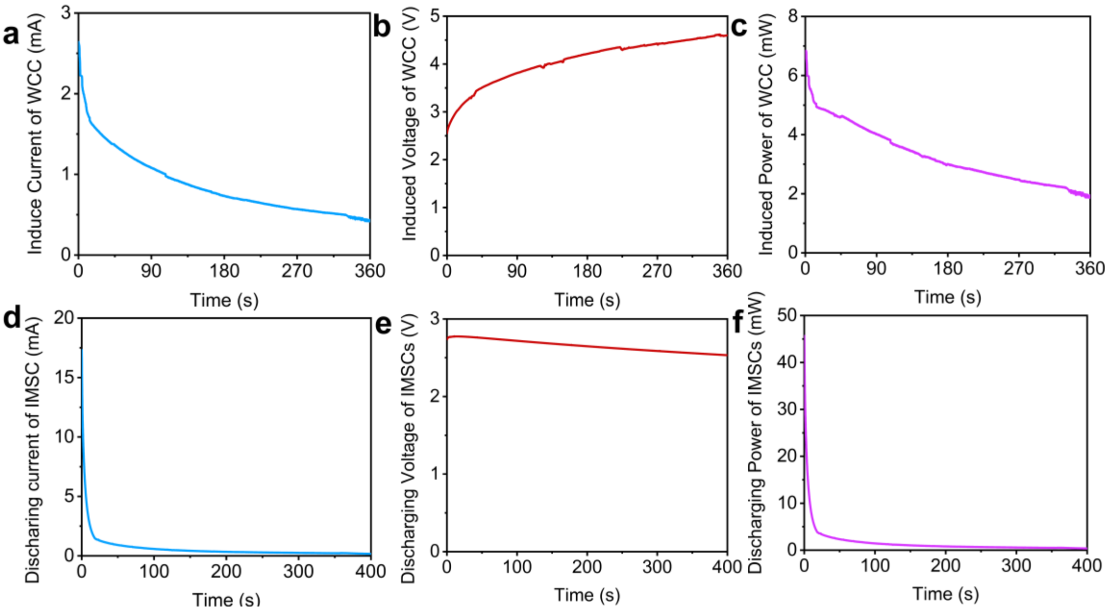

Supplementary Fig. 30 Signals of IMSCs and WCC in the IWC-IMSCs device. a) Induced current, b) voltage, and c) power of WCC in IWC-IMSCs after

rectified during wireless charging. d) Discharging current, e) voltage, and f) power of the IMSCs after wireless charging in the IWC-IMSCs.

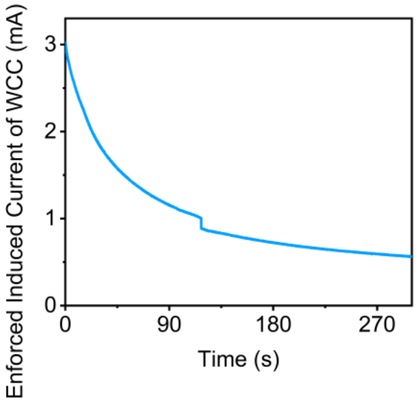

Supplementary Fig. 31 Induced current of WCC in IMSCs when the voltage of transmitting power source is enforced to 12 V. When the voltage improved from 10V to 12V, the charging current will be enforced accordingly and the charging time can be shortened to 5 minutes. The accumulated charge is 310.3 mC.

Supplementary Tables

Supplementary Table 1 Comparison of the areal capacitance of planar micro-supercapacitors

MSC type	Electrode materials	Areal capacitance ($mF\ cm^{-2}$)	Ref
SMSC	$Co(OH)_2$/Pt micro-pillar array	237	s9
SMSC	Carbide-derived carbon/Au film	36	s21
SMSC	PANI nanowire	25.67	s22
SMSC	CoO/CNT nanocomposite	50	s23
SMSC	Laser-scribed graphene/MnO_2	160	s24
SMSC	MnO_2/Au 3D structure	69.15	s18
SMSC-CE	Graphene flake/PEDOT layer	15.3	s25
SMSC-CE	Laser-scribed graphene	2.28	s26
SMSC-CE	Multiple-layer CNTs	4.69	s5
SMSC-CE	Graphene film	9	s27
SMSC-CE	Laser-scribed graphene	0.51	s17
SMSC-CE	Printed graphene/SWCNT	1.324	s7
SMSC-CE	AC/graphite film	454.1	Our
AMSC	GQDs//PANI	0.667	s28
AMSC	MnO_2/rGO/SWCNT	6.439	s29
AMSC	$Ti_3C_2T_x$//Activated carbon	7.8	s30
AMSC	Metal hexacyanoferrate/graphene films	19.84	s31
AMSC	NiCoP@NiOOH//ZIF-C	54.7	s32
AMSC	2D VN and $Co(OH)_2$ nanoflowers	21	s33
AMSC	MnO_2//Ti_3C_2	295	s34
AMSC	Graphene/MnO_2 deposited graphene	400	s35
AMSC	N-GO//N-GO/MoO_2	33.6	s36

SMSC: Symmetric MSC

SMSC-CE: Symmetric MSC made of carbon-based electrodes

AMSC: Asymmetric MSCs

Supplementary Table 2 Comparison of the self-discharging time (SDT) and leakage current of supercapacitors (SC)

Ref	Electrode materials and electrolyte	Initial Voltage (V)	Ending Voltage (V)	SDT (h)	Leakage current (μA)
s37 (MSC)	Graphene, KI/I_2 aqueous solution	1	0.5	3.3	-
s17 (MSC)	Laser-writing rGO, GO	1	0.5	0.8	-
s26 (MSC)	Laser-writing GO film, H_2SO_4	2.5	1.25	13	0.15μA after 12 hours
s38 (MSC)	MnO_2-PPy//V_2O_5-PANI, PVA/H_3PO_4	1.6	0.85	1.1	-
s31 (MSC)	MHG, PVA/LiCl	1.8	0.86	3	-
Our (MSC)	AC/Graphite, ionic liquid	3	1.5	2.3	200μA after 12hours
s39 (SC)	AC, TC/acetonitrile	2	0.9	24	2.2μA after 1.5 hours
s40 (SC)	PPOC, TBAP /propylene carbonate	2	1.44	1	220μA after 18 hours
s41 (SC)	Graphene, Hydroquinone/ H_2SO_4	0.8	0.3	0.4	-

MHG: Metal hexacyanoferrate/graphene hybrid thin films

TC: TEMABF4- 4-n-pentyl-4′-cyanobiphenyl

PPOC: PPO-deposited glassy carbon electrodes

Supplementary References

s1 Shao, Y. *et al.* Design and mechanisms of asymmetric supercapacitors. *Chem. Rev.* **118**, 9233-9280 (2018).

s2 Fan, Z. *et al.* Asymmetric supercapacitors based on graphene/MnO_2 and activated carbon nanofiber electrodes with high power and energy density. *Adv. Funct. Mater.* **21**, 2366-2375 (2011).

s3 Noori, A., El-Kady, M. F., Rahmanifar, M. S., Kaner, R. B. & Mousavi, M. F. Towards establishing standard performance metrics for batteries, supercapacitors and beyond. *Chem. Soc. Rev.* **48**, 1272-1341 (2019).

s4 Katayama, Y., Onodera, H., Yamagata, M. & Miura, T. Electrochemical reduction of oxygen in some hydrophobic room-temperature molten salt systems. *Journal of The Electrochemical Society* **151**, A59 (2004).

s5 Yu, W., Zhou, H., Li, B. Q. & Ding, S. 3D Printing of carbon nanotubes-based microsupercapacitors. *ACS Appl. Mater. Interfaces* **9**, 4597-4604 (2017).

s6 Hota, M. K. *et al.* Integration of electrochemical microsupercapacitors with thin film electronics for on-chip energy storage. *Adv. Mater.* **31**, 1807450 (2019).

s7 Bellani, S. *et al.* Scalable Production of graphene inks via wet-jet milling exfoliation for screen-printed micro-supercapacitors. *Adv. Funct. Mater.* **29**, 1807659 (2019).

s8 Wu, Z.-S. *et al.* Alternating stacked graphene-conducting polymer compact films with ultrahigh areal and volumetric capacitances for high-energy micro-supercapacitors. *Adv. Mater.* **27**, 4054-4061 (2015).

s9 Tian, X. *et al.* Arbitrary shape engineerable spiral micropseudocapacitors with ultrahigh

energy and power densities. *Adv. Mater.* **27**, 7476-7482 (2015).

s10 Wu, Z.-S. *et al.* Layer-by-layer assembled heteroatom-doped graphene films with ultrahigh volumetric capacitance and rate capability for micro-supercapacitors. *Adv. Mater.* **26**, 4552-4558 (2014).

s11 Pang, H., Zhang, Y., Lai, W.-Y., Hu, Z. & Huang, W. Lamellar $K_2Co_3(P_2O_7)_2{\cdot}2H_2O$ nanocrystal whiskers: High-performance flexible all-solid-state asymmetric micro-supercapacitors via inkjet printing. *Nano Energy* **15**, 303-312 (2015).

s12 Zhang, Y. *et al.* All-printed solid-state substrate-versatile and high-performance micro-supercapacitors for in situ fabricated transferable and wearable energy storage via multi-material 3D printing. *J. Power Sources* **403**, 109-117 (2018).

s13 Yanjuan *et al.* Improved conductivity and capacitance of interdigital carbon microelectrodes through integration with carbon nanotubes for micro-supercapacitors. *Nano Research* **9**, 2510-2519 (2016).

s14 Jin, X. *et al.* Flexible and high-performance microsupercapacitors with wide temperature tolerance. *Nano Energy* **64**, 103938 (2019).

s15 Gao, C. *et al.* Versatile origami micro-supercapacitors array as a wind energy harvester. *J. Mater. Chem. A* **6**, 19750-19756 (2018).

s16 Xu, Y. *et al.* Flexible solid-state supercapacitors based on three-dimensional graphene hydrogel films. *ACS Nano* **7**, 4042-4049 (2013).

s17 Gao, W. *et al.* Direct laser writing of micro-supercapacitors on hydrated graphite oxide films. *Nat. Nanotechnol.* **6**, 496-500 (2011).

s18 Li, Y. *et al.* Remarkable improvements in volumetric energy and power of 3D MnO_2

microsupercapacitors by tuning crystallographic structures. *Adv. Funct. Mater.* **26**, 1830-1839 (2016).

s19 Liu, Q. *et al.* The first flexible dual-ion microbattery demonstrates superior capacity and ultrahigh energy density: small and powerful. *Adv. Funct. Mater.* **30**, 2002086 (2020).

s20 Yin, Z. *et al.* Splash-resistant and light-weight silk-sheathed wires for textile electronics. *Nano Lett.* **18**, 7085-7091 (2018).

s21 Chmiola, J., Largeot, C., Taberna, P.-L., Simon, P. & Gogotsi, Y. Monolithic carbide-derived carbon films for micro-supercapacitors. *Science* **328**, 480-483 (2010).

s22 Wang, K. *et al.* An all-solid-state flexible micro-supercapacitor on a chip. *Adv. Ener. Mater.* **1**, 1068-1072 (2011).

s23 Zhu, Y. G., Wang, Y., Shi, Y., Wong, J. I. & Yang, H. Y. CoO nanoflowers woven by CNT network for high energy density flexible micro-supercapacitor. *Nano Energy* **3**, 46-54 (2014).

s24 Hwang, J. Y. *et al.* Direct preparation and processing of graphene/RuO_2 nanocomposite electrodes for high-performance capacitive energy storage. *Nano Energy* **18**, 57-70 (2015).

s25 Lee, H. U. & Kim, S. W. Pen lithography for flexible microsupercapacitors with layer-by-layer assembled graphene flake/PEDOT nanocomposite electrodes. *J. Mater. Chem. A* **5**, 13581-13590 (2017).

s26 El-Kady, M. F. & Kaner, R. B. Scalable fabrication of high-power graphene micro-supercapacitors for flexible and on-chip energy storage. *Nat. Commun.* **4**, 1475 (2013).

s27 Yadav, P., Basu, A., Suryawanshi, A., Game, O. & Ogale, S. Highly stable laser-scribed flexible planar microsupercapacitor using mushroom derived carbon electrodes. *Adv.*

Mater. Inter. **3**, 1600057 (2016).

s28 Liu, W., Yan, X., Chen, J., Feng, Y. & Xue, Q. Novel and high-performance asymmetric micro-supercapacitors based on graphene quantum dots and polyaniline nanofibers. *Nanoscale* **5**, 6053-6062 (2013).

s29 Yu, D. *et al.* Controlled functionalization of carbonaceous fibers for asymmetric solid‐state micro‐supercapacitors with high volumetric energy density. *Adv. Mater.* **26**, 6790-6797 (2014).

s30 Xie, Y. *et al.* High-voltage asymmetric MXene-based on-chip micro-supercapacitors. *Nano Energy* **74**, 104928 (2020).

s31 He, Y. *et al.* Nano-sandwiched metal hexacyanoferrate/graphene hybrid thin films for in-plane asymmetric micro-supercapacitors with ultrahigh energy density. *Mater. Horiz.* **6**, 1041-1049 (2019).

s32 Qiu, M., Sun, P., Cui, G., Tong, Y. & Mai, W. A flexible microsupercapacitor with integral photocatalytic fuel cell for self-charging. *ACS Nano* **13**, 8246-8255 (2019).

s33 Wang, S., Wu, Z. S., Feng, Z., Shi, X. & Bao, X. All-solid-state high-energy planar hybrid micro-supercapacitors based on 2D VN nanosheets and $Co(OH)_2$ nanoflowers. *opj 2D Materials and Applications* **2**, 1-8 (2018).

s34 Zhao, F. *et al.* All two-dimensional pseudocapacitive sheet materials for flexible asymmetric solid-state planar microsupercapacitors with high energy density. *ACS Nano* **14**, 603-610 (2020).

s35 Elkady, M. F. *et al.* Engineering three-dimensional hybrid supercapacitors and microsupercapacitors for high-performance integrated energy storage. *Proc. Natl. Acad.*

Sci. U. S. A. **112**, 4233-4238 (2015).

s36 Zhang, L. *et al.* Shape-tailorable high-energy asymmetric micro-supercapacitors based on plasma reduced and nitrogen-doped graphene oxide and MoO_2 nanoparticles. *J. Mater. Chem. A* **7**, 14328-14336 (2019).

s37 Wu, Z. S., Parvez, K., Feng, X. & Müllen, K. Graphene-based in-plane micro-supercapacitors with high power and energy densities. *Nat. Commun.* **4**, 2487 (2013).

s38 Yue, Y. *et al.* A flexible integrated system containing a microsupercapacitor, a photodetector, and a wireless charging coil. *ACS Nano* **10**, 11249-11257 (2016).

s39 Xia, M., Nie, J., Zhang, Z., Lu, X. & Wang, Z. L. Suppressing self-discharge of supercapacitors via electrorheological effect of liquid crystals. *Nano Energy* **47**, 43-50 (2018).

s40 Tevi, T., Yaghoubi, H., Wang, J. & Takshi, A. Application of poly (p-phenylene oxide) as blocking layer to reduce self-discharge in supercapacitors. *J. Power Sources* **241**, 589-596 (2013).

s41 Chen, L., Bai, H., Huang, Z. & Li, L. Mechanism investigation and suppression of self-discharge in active electrolyte enhanced supercapacitors. *Energy Environ. Sci.* **7**, 1750-1759 (2014).
