# Supplementary material for: A seamlessly integrated device of micro-supercapacitor and wireless charging with ultrahigh energy density and capacitance
Source: Nat Commun. 2021 May 11;12:2647. doi: 10.1038/s41467-021-22912-8 (PMC8113435; doi:10.1038/s41467-021-22912-8)
Supplement: Supplementary file 2 — Description of Additional Supplementary Files [file 41467_2021_22912_MOESM2_ESM.docx]

**Description of Additional Supplementary Files**

**Supplementary Movie 1**

Bending process of the flexibility of the integrated device.

**Supplementary Movie 2**

Entire wireless charging and detecting process to light a red LED.

**Supplementary Movie 3**

An electrical toy car starts running after wireless charging.
