# Supplementary material for: A seamlessly integrated device of micro-supercapacitor and wireless charging with ultrahigh energy density and capacitance
Source: Nat Commun. 2021 May 11;12:2647. doi: 10.1038/s41467-021-22912-8 (PMC8113435; doi:10.1038/s41467-021-22912-8)
Supplement: Supplementary file 6 — Reporting Summary [file 41467_2021_22912_MOESM6_ESM.pdf]

Corresponding author(s): Yang Zhao

Lasing Reporting Summary

Nature Research wishes to improve the reproducibility of the work that we publish. This form is intended for publication with all accepted papers reporting claims of lasing and provides structure for consistency and transparency in reporting. Some list items might not apply to an individual manuscript, but all fields must be completed for clarity.

For further information on Nature Research policies, including our data availability policy, see Authors & Referees.

ü Experimental design

Please check: are the following details reported in the manuscript?

1. Threshold

Plots of device output power versus pump power over a wide range of values indicating a clear threshold

- [ ] Yes
- [x] No

Using the given parameter of the device. Beijing LAJAMIN LASER Co., LM-UVY-5S-Y

2. Linewidth narrowing

Plots of spectral power density for the emission at pump powers below, around, and above the lasing threshold, indicating a clear linewidth narrowing at threshold

- [ ] Yes
- [x] No

Using the given parameter of the device. Beijing LAJAMIN LASER Co., LM-UVY-5S-Y

Resolution of the spectrometer used to make spectral measurements

- [ ] Yes
- [x] No

Using the given parameter of the device. Beijing LAJAMIN LASER Co., LM-UVY-5S-Y

3. Coherent emission

Measurements of the coherence and/or polarization of the emission

- [ ] Yes
- [x] No

Using the given parameter of the device. Beijing LAJAMIN LASER Co., LM-UVY-5S-Y

4. Beam spatial profile

Image and/or measurement of the spatial shape and profile of the emission, showing a well-defined beam above threshold

- [ ] Yes
- [x] No

Using the given parameter of the device. Beijing LAJAMIN LASER Co., LM-UVY-5S-Y

5. Operating conditions

Description of the laser and pumping conditions
Continuous-wave, pulsed, temperature of operation

- [x] Yes
- [ ] No

In the Characterization measurements of Methods.

Threshold values provided as density values (e.g. W cm^{-2} or J cm^{-2}) taking into account the area of the device

- [x] Yes
- [ ] No

In the Characterization measurements of Methods.

6. Alternative explanations

Reasoning as to why alternative explanations have been ruled out as responsible for the emission characteristics
e.g. amplified spontaneous, directional scattering; modification of fluorescence spectrum by the cavity

- [ ] Yes
- [x] No

Using the given parameter of the device. Beijing LAJAMIN LASER Co., LM-UVY-5S-Y. No modification of emission characteristics has been put forward for the good performance devices.

7. Theoretical analysis

Theoretical analysis that ensures that the experimental values measured are realistic and reasonable
e.g. laser threshold, linewidth, cavity gain-loss, efficiency

- [ ] Yes
- [x] No

We have fabricated a large number of devices with good performance under stable (single) and simple laser parameters. No obvious difference between devices has been found. The stable laser parameters and consistency of devices guaranteed the reliability of the data. So no strict requests for the device to do some theoretical analysis.

8. Statistics

Number of devices fabricated and tested

- [ ] Yes
- [x] No

Owing to the reliable and stable performance of the device, we use a series fabricated devices to test, and there are no obvious difference between them, so there is no strict requests from statistics.

Statistical analysis of the device performance and lifetime (time to failure)

☐ Yes
☒ No

The device remains good performance after 3000 cycles, about 5 days, so there is no statistical analysis.
